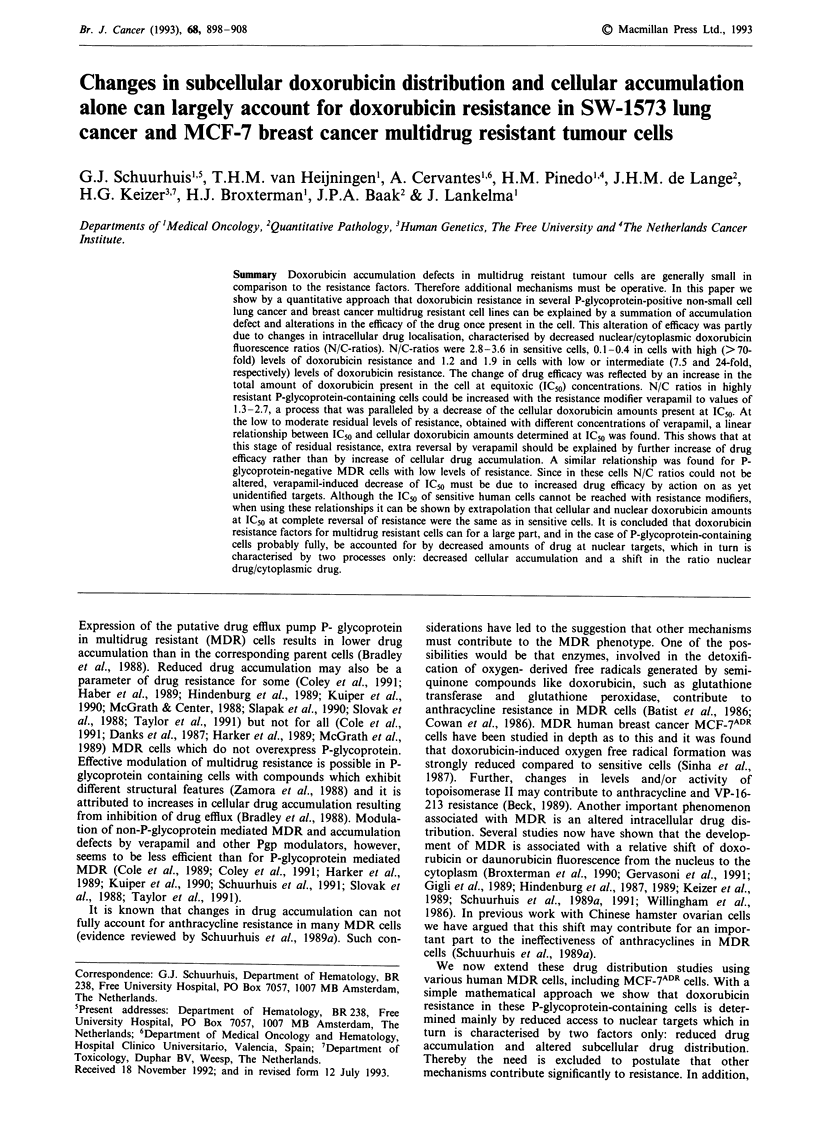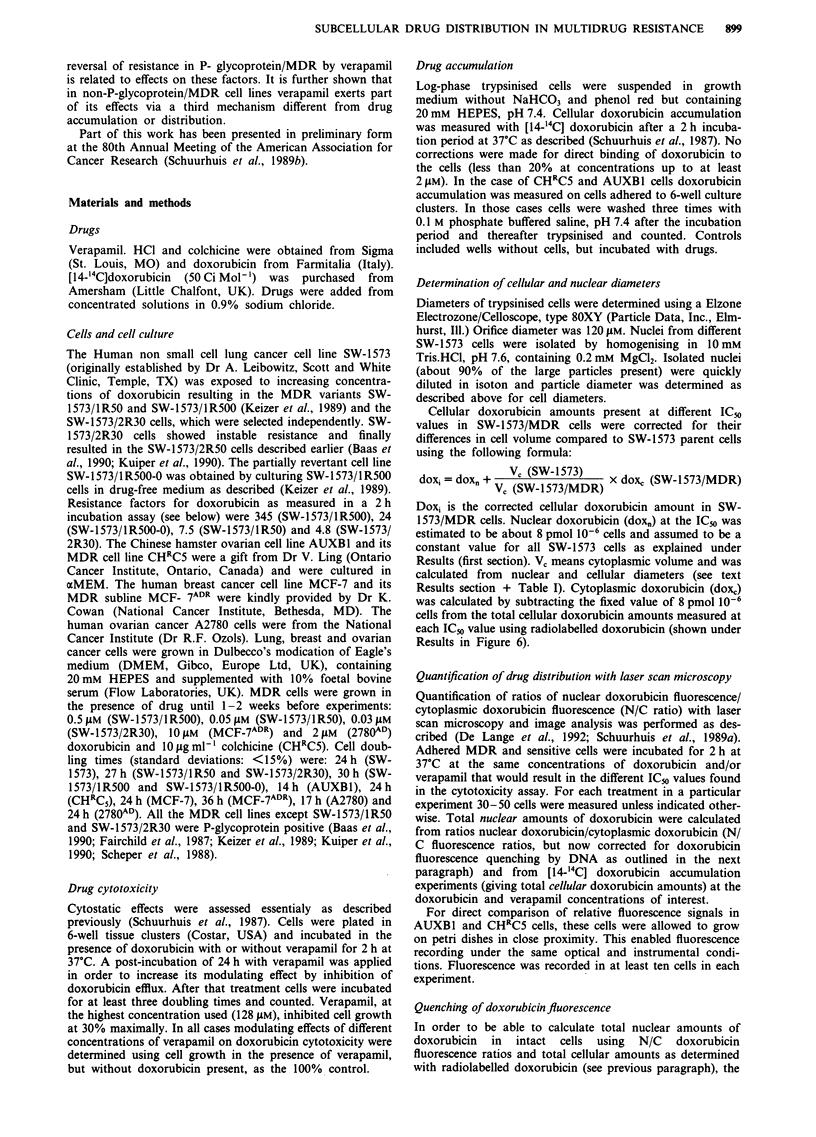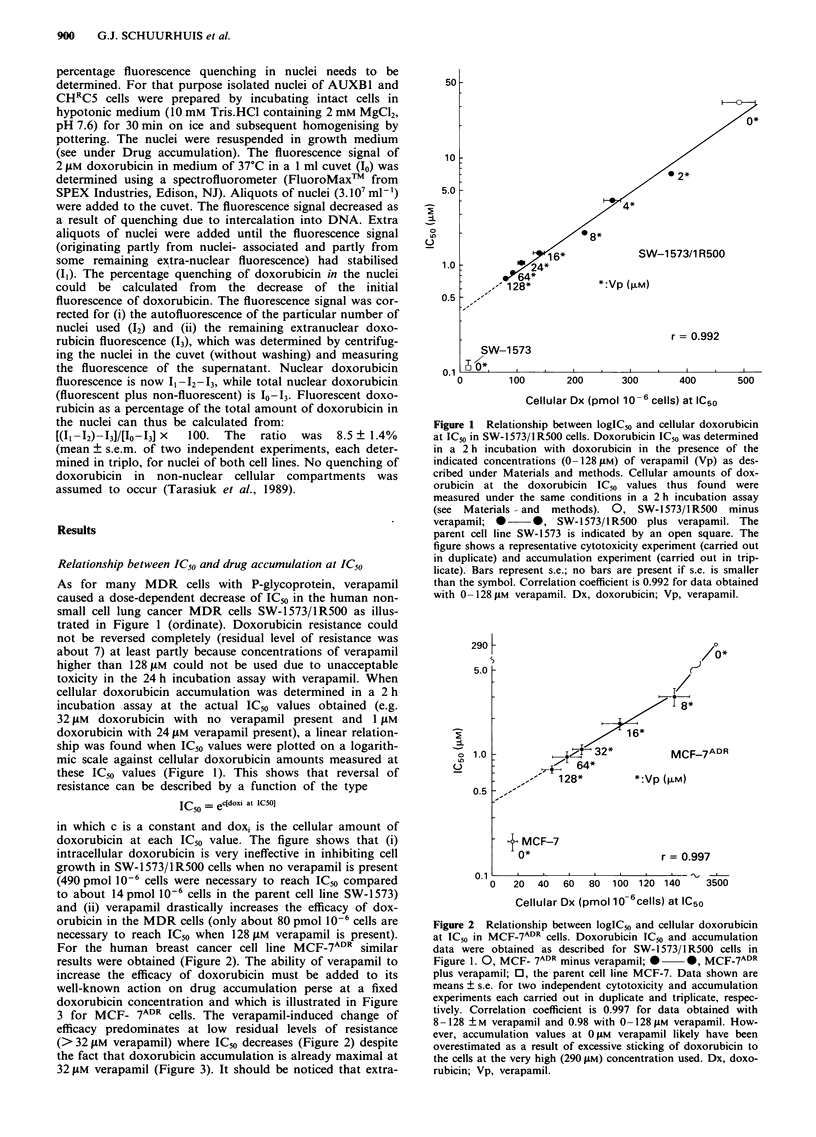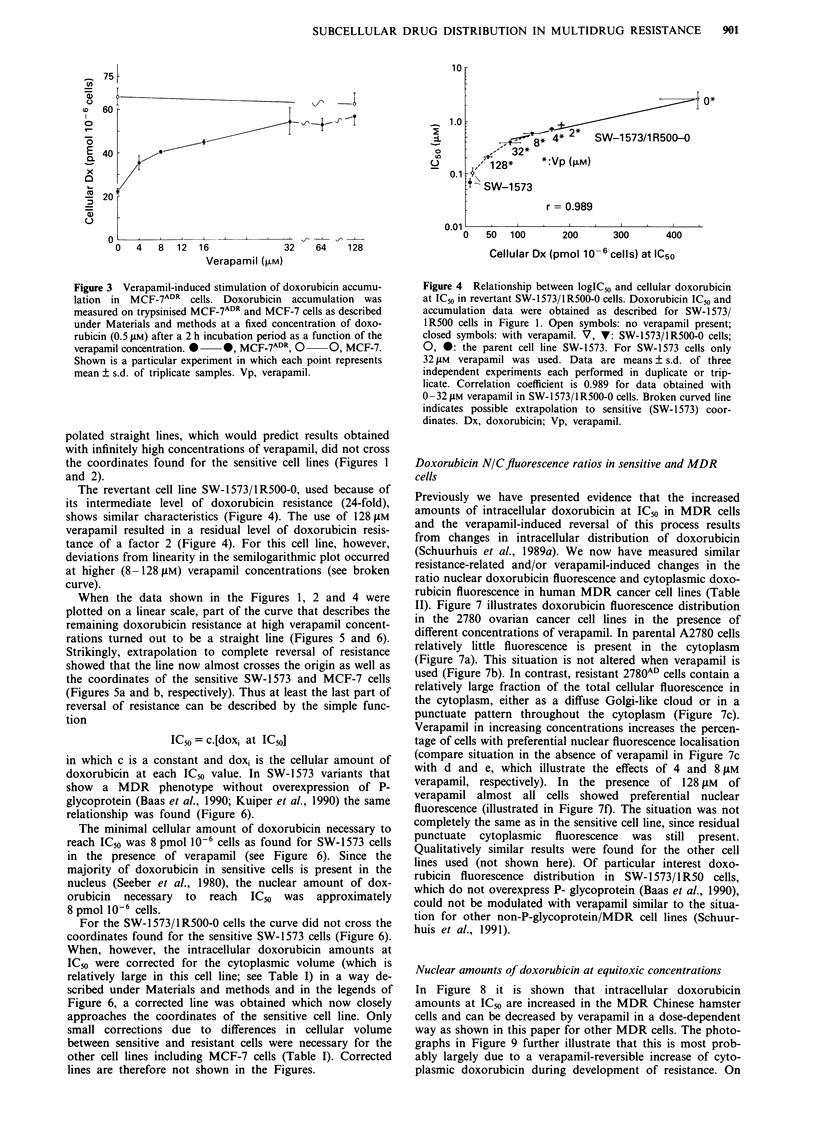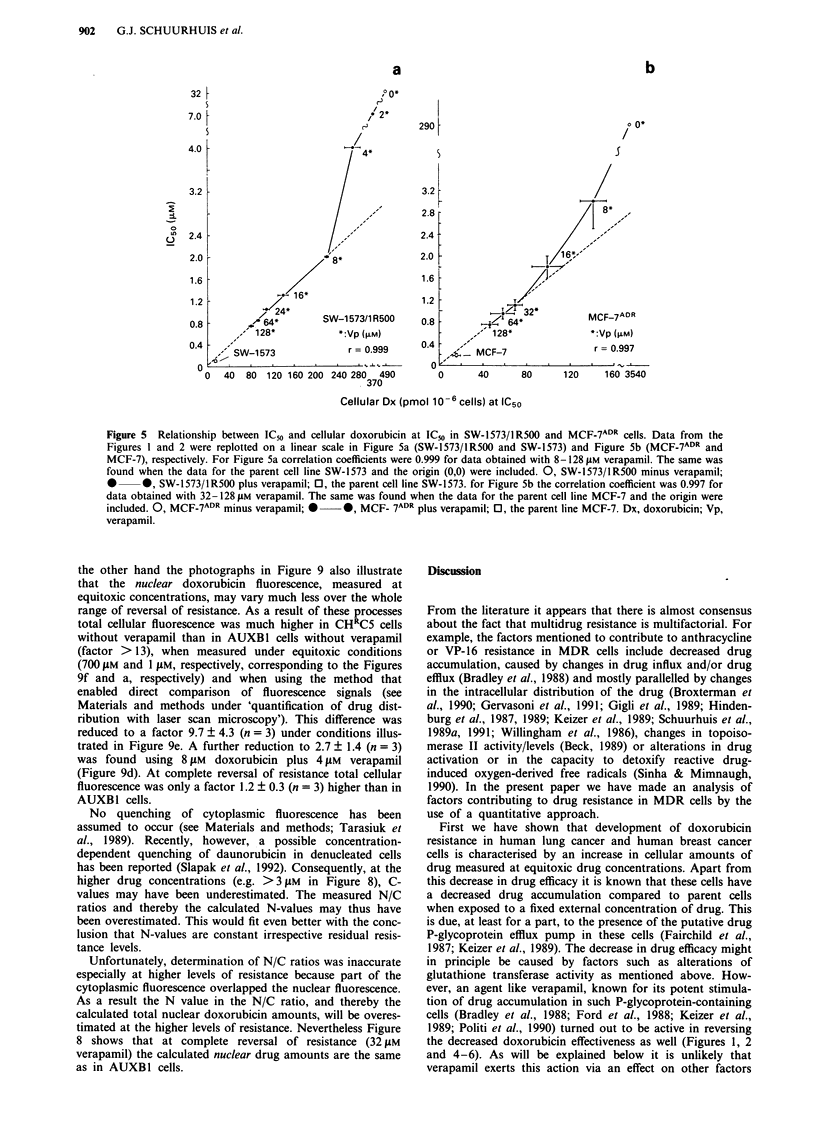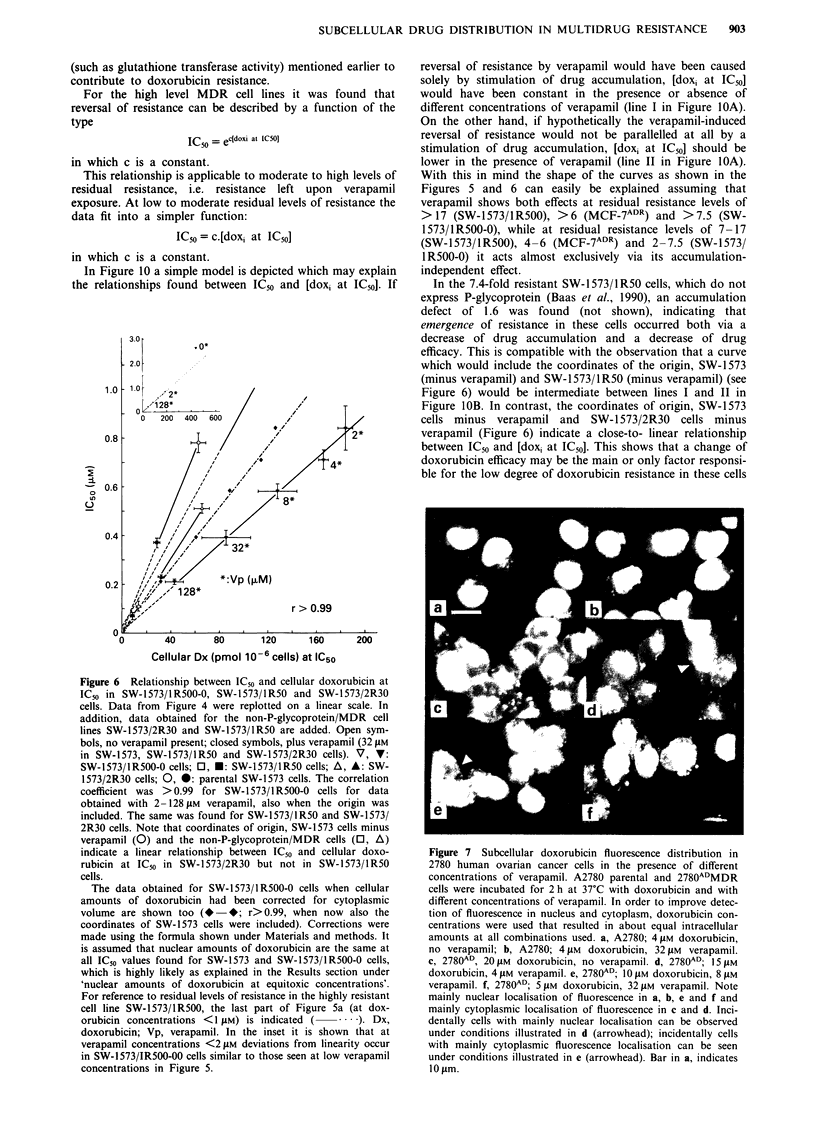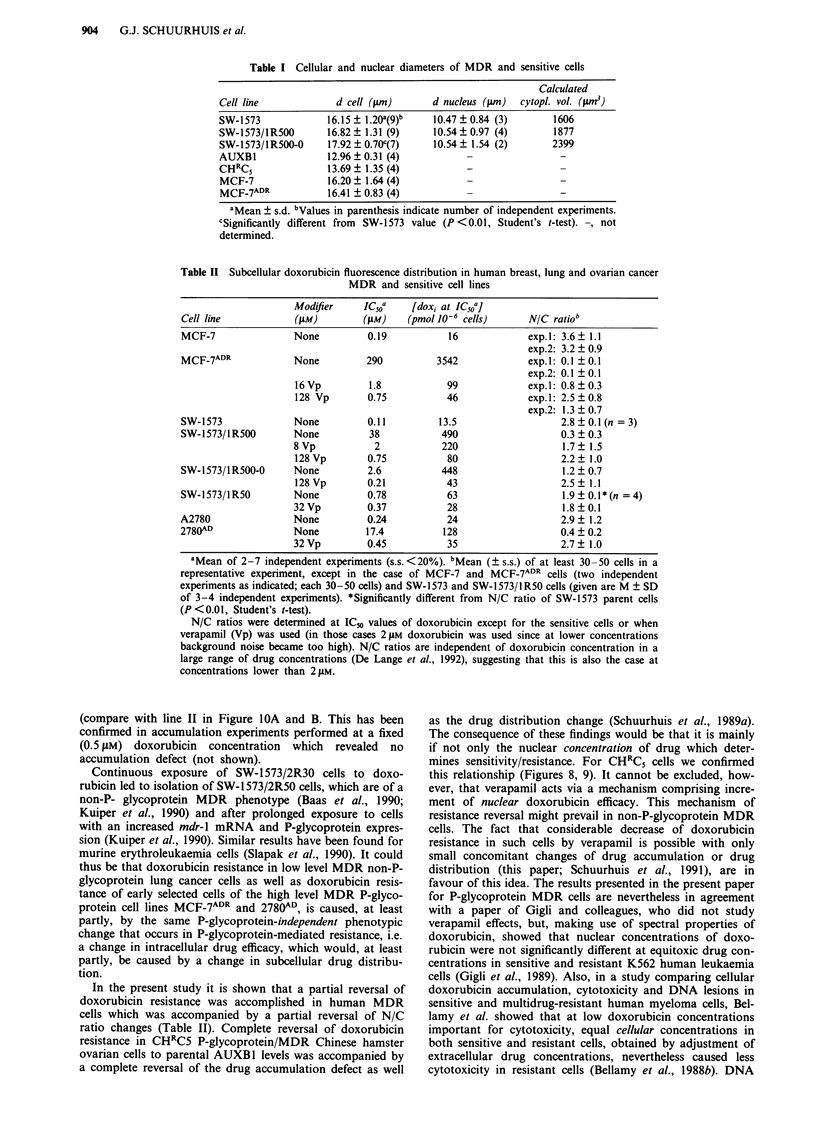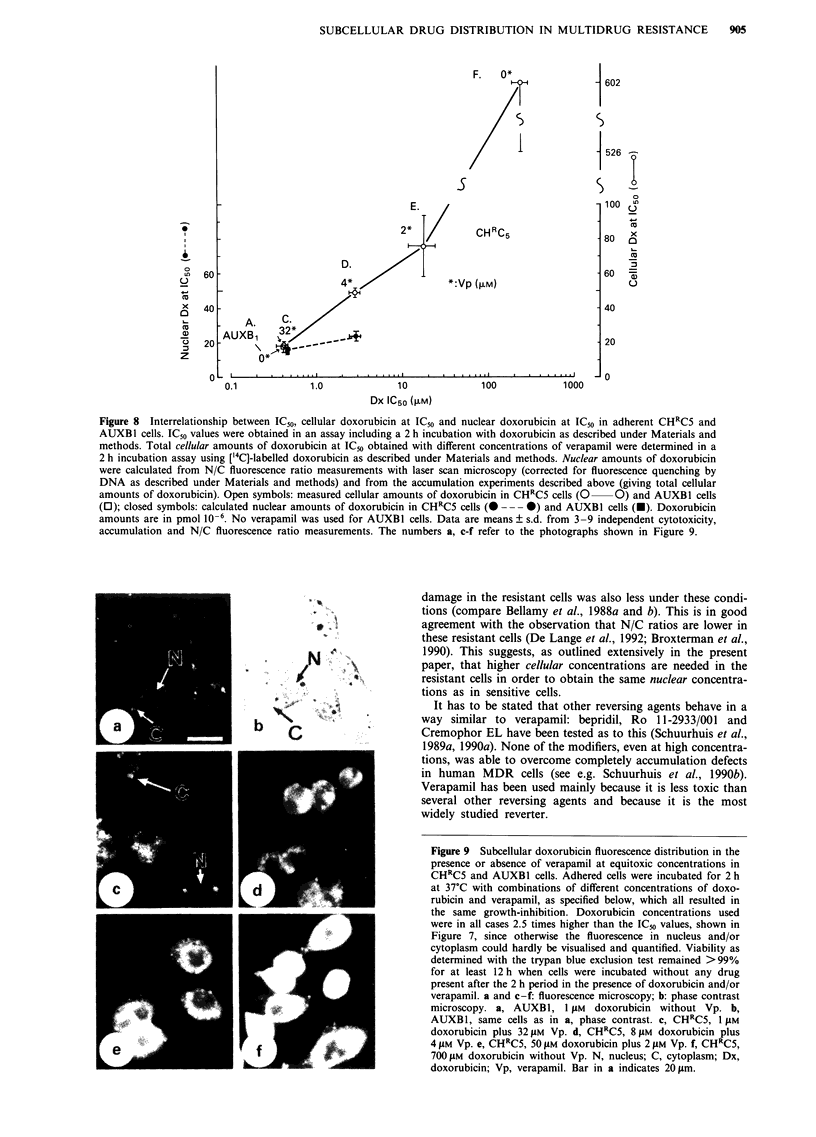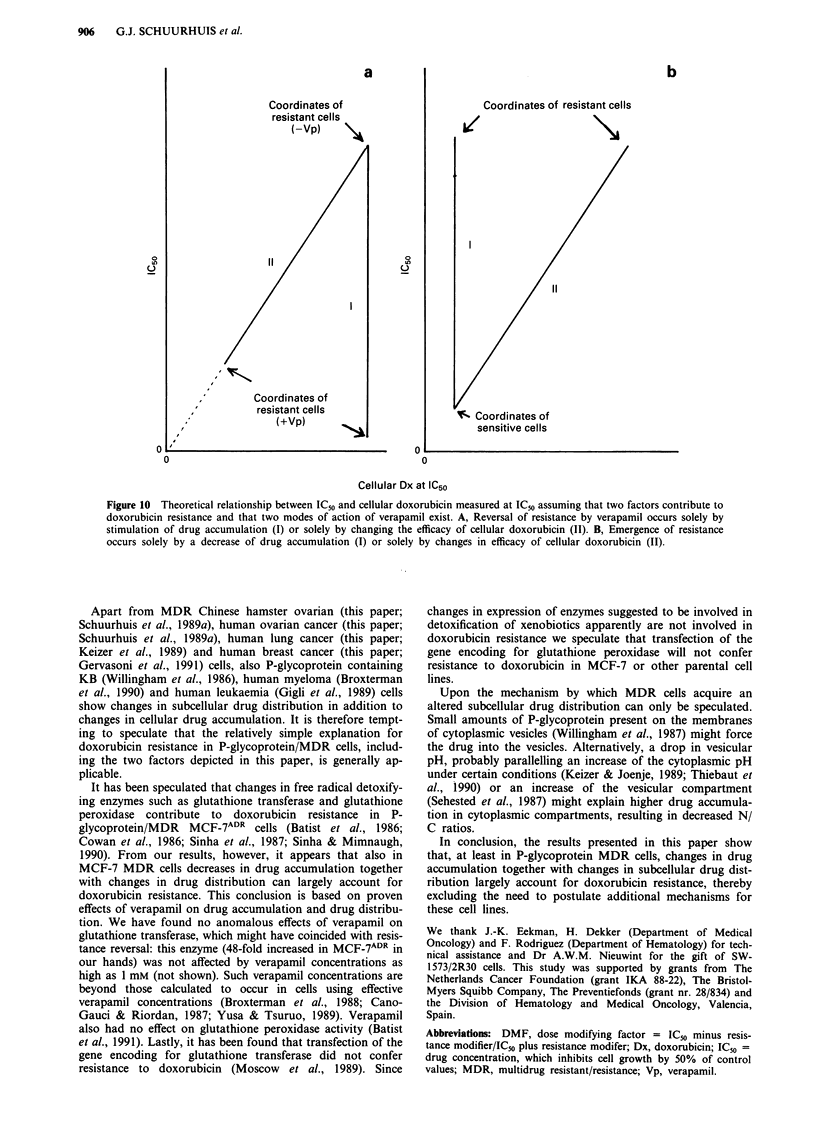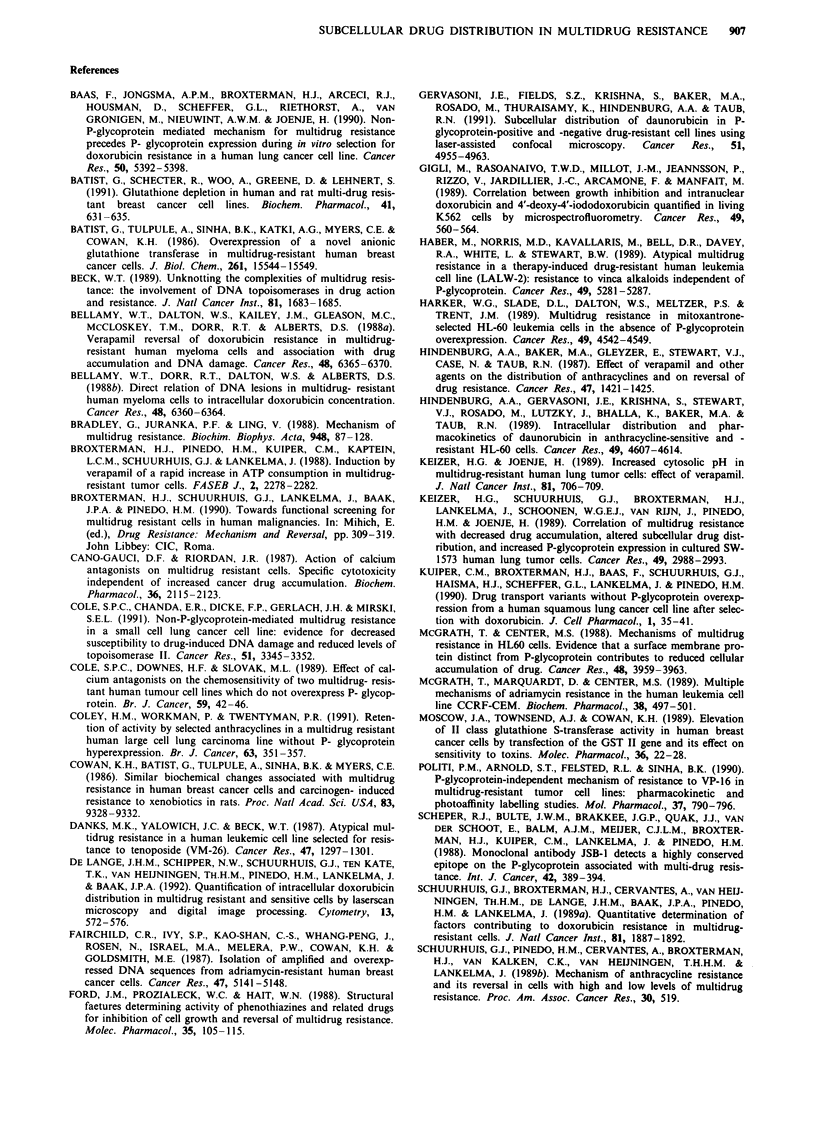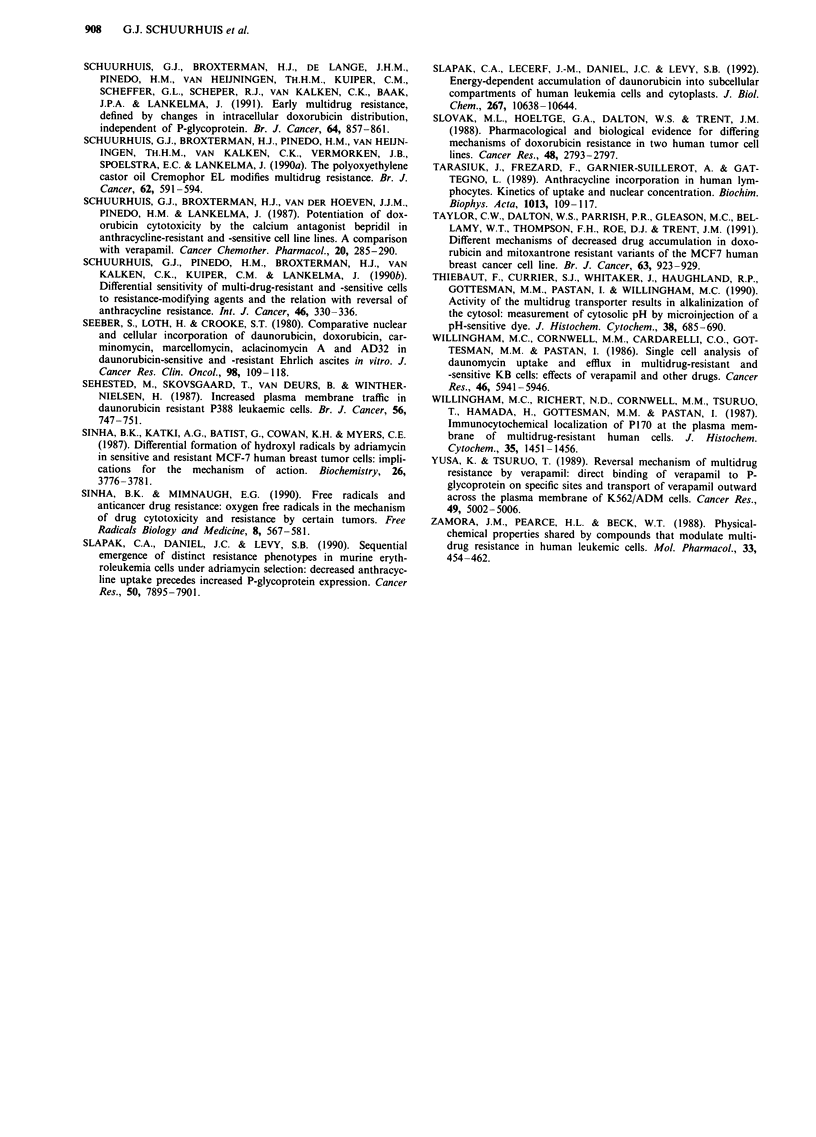# Changes in subcellular doxorubicin distribution and cellular accumulation alone can largely account for doxorubicin resistance in SW-1573 lung cancer and MCF-7 breast cancer multidrug resistant tumour cells.

**DOI:** 10.1038/bjc.1993.452

**Published:** 1993-11

**Authors:** G. J. Schuurhuis, T. H. van Heijningen, A. Cervantes, H. M. Pinedo, J. H. de Lange, H. G. Keizer, H. J. Broxterman, J. P. Baak, J. Lankelma

**Affiliations:** Department of Medical Oncology, Free University, The Netherlands.

## Abstract

**Images:**


Br. J. Cancer (1993), 68, 898–908 © Macmillan Press Ltd., 1993

### Changes in subcellular doxorubicin distribution and cellular accumulation alone can largely account for doxorubicin resistance in SW-1573 lung cancer and MCF-7 breast cancer multidrug resistant tumour cells

G.J. Schuurhuis[1,5], T.H.M. van Heijningen[1], A. Cervantes[1,6], H.M. Pinedo[1,4], J.H.M. de Lange[2], H.G. Keizer[3,7], H.J. Broxterman[1], J.P.A. Baak[2] & J. Lankelma[1]

*Departments of [1]Medical Oncology, [2]Quantitative Pathology, [3]Human Genetics, The Free University and [4]The Netherlands Cancer Institute.*

**Summary** Doxorubicin accumulation defects in multidrug reistant tumour cells are generally small in comparison to the resistance factors. Therefore additional mechanisms must be operative. In this paper we show by a quantitative approach that doxorubicin resistance in several P-glycoprotein-positive non-small cell lung cancer and breast cancer multidrug resistant cell lines can be explained by a summation of accumulation defect and alterations in the efficacy of the drug once present in the cell. This alteration of efficacy was partly due to changes in intracellular drug localisation, characterised by decreased nuclear/cytoplasmic doxorubicin fluorescence ratios (N/C-ratios). N/C-ratios were 2.8–3.6 in sensitive cells, 0.1–0.4 in cells with high (>70-fold) levels of doxorubicin resistance and 1.2 and 1.9 in cells with low or intermediate (7.5 and 24-fold, respectively) levels of doxorubicin resistance. The change of drug efficacy was reflected by an increase in the total amount of doxorubicin present in the cell at equitoxic ($IC_{50}$) concentrations. N/C ratios in highly resistant P-glycoprotein-containing cells could be increased with the resistance modifier verapamil to values of 1.3–2.7, a process that was paralleled by a decrease of the cellular doxorubicin amounts present at $IC_{50}$. At the low to moderate residual levels of resistance, obtained with different concentrations of verapamil, a linear relationship between $IC_{50}$ and cellular doxorubicin amounts determined at $IC_{50}$ was found. This shows that at this stage of residual resistance, extra reversal by verapamil should be explained by further increase of drug efficacy rather than by increase of cellular drug accumulation. A similar relationship was found for P-glycoprotein-negative MDR cells with low levels of resistance. Since in these cells N/C ratios could not be altered, verapamil-induced decrease of $IC_{50}$ must be due to increased drug efficacy by action on as yet unidentified targets. Although the $IC_{50}$ of sensitive human cells cannot be reached with resistance modifiers, when using these relationships it can be shown by extrapolation that cellular and nuclear doxorubicin amounts at $IC_{50}$ at complete reversal of resistance were the same as in sensitive cells. It is concluded that doxorubicin resistance factors for multidrug resistant cells can for a large part, and in the case of P-glycoprotein-containing cells probably fully, be accounted for by decreased amounts of drug at nuclear targets, which in turn is characterised by two processes only: decreased cellular accumulation and a shift in the ratio nuclear drug/cytoplasmic drug.

Expression of the putative drug efflux pump P- glycoprotein in multidrug resistant (MDR) cells results in lower drug accumulation than in the corresponding parent cells (Bradley *et al.*, 1988). Reduced drug accumulation may also be a parameter of drug resistance for some (Coley *et al.*, 1991; Haber *et al.*, 1989; Hindenburg *et al.*, 1989; Kuiper *et al.*, 1990; McGrath & Center, 1988; Slapak *et al.*, 1990; Slovak *et al.*, 1988; Taylor *et al.*, 1991) but not for all (Cole *et al.*, 1991; Danks *et al.*, 1987; Harker *et al.*, 1989; McGrath *et al.*, 1989) MDR cells which do not overexpress P-glycoprotein. Effective modulation of multidrug resistance is possible in P-glycoprotein containing cells with compounds which exhibit different structural features (Zamora *et al.*, 1988) and it is attributed to increases in cellular drug accumulation resulting from inhibition of drug efflux (Bradley *et al.*, 1988). Modulation of non-P-glycoprotein mediated MDR and accumulation defects by verapamil and other Pgp modulators, however, seems to be less efficient than for P-glycoprotein mediated MDR (Cole *et al.*, 1989; Coley *et al.*, 1991; Harker *et al.*, 1989; Kuiper *et al.*, 1990; Schuurhuis *et al.*, 1991; Slovak *et al.*, 1988; Taylor *et al.*, 1991).

It is known that changes in drug accumulation can not fully account for anthracycline resistance in many MDR cells (evidence reviewed by Schuurhuis *et al.*, 1989*a*). Such considerations have led to the suggestion that other mechanisms must contribute to the MDR phenotype. One of the possibilities would be that enzymes, involved in the detoxification of oxygen- derived free radicals generated by semi-quinone compounds like doxorubicin, such as glutathione transferase and glutathione peroxidase, contribute to anthracycline resistance in MDR cells (Batist *et al.*, 1986; Cowan *et al.*, 1986). MDR human breast cancer MCF-7$^{ADR}$ cells have been studied in depth as to this and it was found that doxorubicin-induced oxygen free radical formation was strongly reduced compared to sensitive cells (Sinha *et al.*, 1987). Further, changes in levels and/or activity of topoisomerase II may contribute to anthracycline and VP-16-213 resistance (Beck, 1989). Another important phenomenon associated with MDR is an altered intracellular drug distribution. Several studies now have shown that the development of MDR is associated with a relative shift of doxorubicin or daunorubicin fluorescence from the nucleus to the cytoplasm (Broxterman *et al.*, 1990; Gervasoni *et al.*, 1991; Gigli *et al.*, 1989; Hindenburg *et al.*, 1987, 1989; Keizer *et al.*, 1989; Schuurhuis *et al.*, 1989*a*, 1991; Willingham *et al.*, 1986). In previous work with Chinese hamster ovarian cells we have argued that this shift may contribute for an important part to the ineffectiveness of anthracyclines in MDR cells (Schuurhuis *et al.*, 1989*a*).

We now extend these drug distribution studies using various human MDR cells, including MCF-7$^{ADR}$ cells. With a simple mathematical approach we show that doxorubicin resistance in these P-glycoprotein-containing cells is determined mainly by reduced access to nuclear targets which in turn is characterised by two factors only: reduced drug accumulation and altered subcellular drug distribution. Thereby the need is excluded to postulate that other mechanisms contribute significantly to resistance. In addition,

Correspondence: G.J. Schuurhuis, Department of Hematology, BR 238, Free University Hospital, PO Box 7057, 1007 MB Amsterdam, The Netherlands.

[5]Present addresses: Department of Hematology, BR 238, Free University Hospital, PO Box 7057, 1007 MB Amsterdam, The Netherlands; [6]Department of Medical Oncology and Hematology, Hospital Clinico Universitario, Valencia, Spain; [7]Department of Toxicology, Duphar BV, Weesp, The Netherlands.

Received 18 November 1992; and in revised form 12 July 1993.

reversal of resistance in P- glycoprotein/MDR by verapamil is related to effects on these factors. It is further shown that in non-P-glycoprotein/MDR cell lines verapamil exerts part of its effects via a third mechanism different from drug accumulation or distribution.

Part of this work has been presented in preliminary form at the 80th Annual Meeting of the American Association for Cancer Research (Schuurhuis *et al.*, 1989*b*).

### Materials and methods

### *Drugs*

Verapamil. HCl and colchicine were obtained from Sigma (St. Louis, MO) and doxorubicin from Farmitalia (Italy). [14-$^{14}$C]doxorubicin (50 Ci $Mol^{-1}$) was purchased from Amersham (Little Chalfont, UK). Drugs were added from concentrated solutions in 0.9% sodium chloride.

### *Cells and cell culture*

The Human non small cell lung cancer cell line SW-1573 (originally established by Dr A. Leibowitz, Scott and White Clinic, Temple, TX) was exposed to increasing concentrations of doxorubicin resulting in the MDR variants SW-1573/1R50 and SW-1573/1R500 (Keizer *et al.*, 1989) and the SW-1573/2R30 cells, which were selected independently. SW-1573/2R30 cells showed instable resistance and finally resulted in the SW-1573/2R50 cells described earlier (Baas *et al.*, 1990; Kuiper *et al.*, 1990). The partially revertant cell line SW-1573/1R500-0 was obtained by culturing SW-1573/1R500 cells in drug-free medium as described (Keizer *et al.*, 1989). Resistance factors for doxorubicin as measured in a 2 h incubation assay (see below) were 345 (SW-1573/1R500), 24 (SW-1573/1R500-0), 7.5 (SW-1573/1R50) and 4.8 (SW-1573/2R30). The Chinese hamster ovarian cell line AUXB1 and its MDR cell line $CH^RC5$ were a gift from Dr V. Ling (Ontario Cancer Institute, Ontario, Canada) and were cultured in αMEM. The human breast cancer cell line MCF-7 and its MDR subline MCF- $7^{ADR}$ were kindly provided by Dr K. Cowan (National Cancer Institute, Bethesda, MD). The human ovarian cancer A2780 cells were from the National Cancer Institute (Dr R.F. Ozols). Lung, breast and ovarian cancer cells were grown in Dulbecco's modication of Eagle's medium (DMEM, Gibco, Europe Ltd, UK), containing 20 mM HEPES and supplemented with 10% foetal bovine serum (Flow Laboratories, UK). MDR cells were grown in the presence of drug until 1–2 weeks before experiments: 0.5 μM (SW-1573/1R500), 0.05 μM (SW-1573/1R50), 0.03 μM (SW-1573/2R30), 10 μM (MCF-$7^{ADR}$) and 2 μM ($2780^{AD}$) doxorubicin and 10 μg $ml^{-1}$ colchicine ($CH^RC5$). Cell doubling times (standard deviations: <15%) were: 24 h (SW-1573), 27 h (SW-1573/1R50 and SW-1573/2R30), 30 h (SW-1573/1R500 and SW-1573/1R500-0), 14 h (AUXB1), 24 h ($CH^RC_5$), 24 h (MCF-7), 36 h (MCF-$7^{ADR}$), 17 h (A2780) and 24 h ($2780^{AD}$). All the MDR cell lines except SW-1573/1R50 and SW-1573/2R30 were P-glycoprotein positive (Baas *et al.*, 1990; Fairchild *et al.*, 1987; Keizer *et al.*, 1989; Kuiper *et al.*, 1990; Scheper *et al.*, 1988).

### *Drug cytotoxicity*

Cytostatic effects were assessed essentialy as described previously (Schuurhuis *et al.*, 1987). Cells were plated in 6-well tissue clusters (Costar, USA) and incubated in the presence of doxorubicin with or without verapamil for 2 h at 37°C. A post-incubation of 24 h with verapamil was applied in order to increase its modulating effect by inhibition of doxorubicin efflux. After that treatment cells were incubated for at least three doubling times and counted. Verapamil, at the highest concentration used (128 μM), inhibited cell growth at 30% maximally. In all cases modulating effects of different concentrations of verapamil on doxorubicin cytotoxicity were determined using cell growth in the presence of verapamil, but without doxorubicin present, as the 100% control.

### *Drug accumulation*

Log-phase trypsinised cells were suspended in growth medium without $NaHCO_3$ and phenol red but containing 20 mM HEPES, pH 7.4. Cellular doxorubicin accumulation was measured with [14-$^{14}$C] doxorubicin after a 2 h incubation period at 37°C as described (Schuurhuis *et al.*, 1987). No corrections were made for direct binding of doxorubicin to the cells (less than 20% at concentrations up to at least 2 μM). In the case of $CH^RC5$ and AUXB1 cells doxorubicin accumulation was measured on cells adhered to 6-well culture clusters. In those cases cells were washed three times with 0.1 M phosphate buffered saline, pH 7.4 after the incubation period and thereafter trypsinised and counted. Controls included wells without cells, but incubated with drugs.

### *Determination of cellular and nuclear diameters*

Diameters of trypsinised cells were determined using a Elzone Electrozone/Celloscope, type 80XY (Particle Data, Inc., Elmhurst, Ill.) Orifice diameter was 120 μM. Nuclei from different SW-1573 cells were isolated by homogenising in 10 mM Tris.HCl, pH 7.6, containing 0.2 mM $MgCl_2$. Isolated nuclei (about 90% of the large particles present) were quickly diluted in isoton and particle diameter was determined as described above for cell diameters.

Cellular doxorubicin amounts present at different $IC_{50}$ values in SW-1573/MDR cells were corrected for their differences in cell volume compared to SW-1573 parent cells using the following formula:

$$dox_i = dox_n + \frac{V_c\ (SW\text{-}1573)}{V_c\ (SW\text{-}1573/MDR)} \times dox_c\ (SW\text{-}1573/MDR)$$

$Dox_i$ is the corrected cellular doxorubicin amount in SW-1573/MDR cells. Nuclear doxorubicin ($dox_n$) at the $IC_{50}$ was estimated to be about 8 pmol $10^{-6}$ cells and assumed to be a constant value for all SW-1573 cells as explained under Results (first section). $V_c$ means cytoplasmic volume and was calculated from nuclear and cellular diameters (see text Results section + Table I). Cytoplasmic doxorubicin ($dox_c$) was calculated by subtracting the fixed value of 8 pmol $10^{-6}$ cells from the total cellular doxorubicin amounts measured at each $IC_{50}$ value using radiolabelled doxorubicin (shown under Results in Figure 6).

### *Quantification of drug distribution with laser scan microscopy*

Quantification of ratios of nuclear doxorubicin fluorescence/cytoplasmic doxorubicin fluorescence (N/C ratio) with laser scan microscopy and image analysis was performed as described (De Lange *et al.*, 1992; Schuurhuis *et al.*, 1989*a*). Adhered MDR and sensitive cells were incubated for 2 h at 37°C at the same concentrations of doxorubicin and/or verapamil that would result in the different $IC_{50}$ values found in the cytotoxicity assay. For each treatment in a particular experiment 30–50 cells were measured unless indicated otherwise. Total *nuclear* amounts of doxorubicin were calculated from ratios nuclear doxorubicin/cytoplasmic doxorubicin (N/C fluorescence ratios, but now corrected for doxorubicin fluorescence quenching by DNA as outlined in the next paragraph) and from [14-$^{14}$C] doxorubicin accumulation experiments (giving total *cellular* doxorubicin amounts) at the doxorubicin and verapamil concentrations of interest.

For direct comparison of relative fluorescence signals in AUXB1 and $CH^RC5$ cells, these cells were allowed to grow on petri dishes in close proximity. This enabled fluorescence recording under the same optical and instrumental conditions. Fluorescence was recorded in at least ten cells in each experiment.

### *Quenching of doxorubicin fluorescence*

In order to be able to calculate total nuclear amounts of doxorubicin in intact cells using N/C doxorubicin fluorescence ratios and total cellular amounts as determined with radiolabelled doxorubicin (see previous paragraph), the percentage fluorescence quenching in nuclei needs to be determined. For that purpose isolated nuclei of AUXB1 and $CH^RC5$ cells were prepared by incubating intact cells in hypotonic medium (10 mM Tris.HCl containing 2 mM $MgCl_2$, pH 7.6) for 30 min on ice and subsequent homogenising by pottering. The nuclei were resuspended in growth medium (see under Drug accumulation). The fluorescence signal of 2 $\mu$M doxorubicin in medium of 37°C in a 1 ml cuvet ($I_0$) was determined using a spectrofluorometer (FluoroMax™ from SPEX Industries, Edison, NJ). Aliquots of nuclei ($3.10^7$ $ml^{-1}$) were added to the cuvet. The fluorescence signal decreased as a result of quenching due to intercalation into DNA. Extra aliquots of nuclei were added until the fluorescence signal (originating partly from nuclei- associated and partly from some remaining extra-nuclear fluorescence) had stabilised ($I_1$). The percentage quenching of doxorubicin *in* the nuclei could be calculated from the decrease of the initial fluorescence of doxorubicin. The fluorescence signal was corrected for (i) the autofluorescence of the particular number of nuclei used ($I_2$) and (ii) the remaining extranuclear doxorubicin fluorescence ($I_3$), which was determined by centrifuging the nuclei in the cuvet (without washing) and measuring the fluorescence of the supernatant. Nuclear doxorubicin fluorescence is now $I_1-I_2-I_3$, while total nuclear doxorubicin (fluorescent plus non-fluorescent) is $I_0-I_3$. Fluorescent doxorubicin as a percentage of the total amount of doxorubicin in the nuclei can thus be calculated from: $[(I_1-I_2)-I_3]/[I_0-I_3] \times 100$. The ratio was $8.5 \pm 1.4\%$ (mean ± s.e.m. of two independent experiments, each determined in triplo, for nuclei of both cell lines. No quenching of doxorubicin in non-nuclear cellular compartments was assumed to occur (Tarasiuk *et al.*, 1989).

### Results

### *Relationship between $IC_{50}$ and drug accumulation at $IC_{50}$*

As for many MDR cells with P-glycoprotein, verapamil caused a dose-dependent decrease of $IC_{50}$ in the human non-small cell lung cancer MDR cells SW-1573/1R500 as illustrated in Figure 1 (ordinate). Doxorubicin resistance could not be reversed completely (residual level of resistance was about 7) at least partly because concentrations of verapamil higher than 128 $\mu$M could not be used due to unacceptable toxicity in the 24 h incubation assay with verapamil. When cellular doxorubicin accumulation was determined in a 2 h incubation assay at the actual $IC_{50}$ values obtained (e.g. 32 $\mu$M doxorubicin with no verapamil present and 1 $\mu$M doxorubicin with 24 $\mu$M verapamil present), a linear relationship was found when $IC_{50}$ values were plotted on a logarithmic scale against cellular doxorubicin amounts measured at these $IC_{50}$ values (Figure 1). This shows that reversal of resistance can be described by a function of the type

$$IC_{50} = e^{c[\text{doxi at IC50}]}$$

in which c is a constant and $dox_i$ is the cellular amount of doxorubicin at each $IC_{50}$ value. The figure shows that (i) intracellular doxorubicin is very ineffective in inhibiting cell growth in SW-1573/1R500 cells when no verapamil is present (490 pmol $10^{-6}$ cells were necessary to reach $IC_{50}$ compared to about 14 pmol $10^{-6}$ cells in the parent cell line SW-1573) and (ii) verapamil drastically increases the efficacy of doxorubicin in the MDR cells (only about 80 pmol $10^{-6}$ cells are necessary to reach $IC_{50}$ when 128 $\mu$M verapamil is present). For the human breast cancer cell line MCF-$7^{ADR}$ similar results were obtained (Figure 2). The ability of verapamil to increase the efficacy of doxorubicin must be added to its well-known action on drug accumulation perse at a fixed doxorubicin concentration and which is illustrated in Figure 3 for MCF- $7^{ADR}$ cells. The verapamil-induced change of efficacy predominates at low residual levels of resistance (> 32 $\mu$M verapamil) where $IC_{50}$ decreases (Figure 2) despite the fact that doxorubicin accumulation is already maximal at 32 $\mu$M verapamil (Figure 3). It should be noticed that extra-

**Figure 1** Relationship between $\log IC_{50}$ and cellular doxorubicin at $IC_{50}$ in SW-1573/1R500 cells. Doxorubicin $IC_{50}$ was determined in a 2 h incubation with doxorubicin in the presence of the indicated concentrations (0–128 $\mu$M) of verapamil (Vp) as described under Materials and methods. Cellular amounts of doxorubicin at the doxorubicin $IC_{50}$ values thus found were measured under the same conditions in a 2 h incubation assay (see Materials and methods). ○, SW-1573/1R500 minus verapamil; ●——●, SW-1573/1R500 plus verapamil. The parent cell line SW-1573 is indicated by an open square. The figure shows a representative cytotoxicity experiment (carried out in duplicate) and accumulation experiment (carried out in triplicate). Bars represent s.e.; no bars are present if s.e. is smaller than the symbol. Correlation coefficient is 0.992 for data obtained with 0–128 $\mu$M verapamil. Dx, doxorubicin; Vp, verapamil.

**Figure 2** Relationship between $\log IC_{50}$ and cellular doxorubicin at $IC_{50}$ in MCF-$7^{ADR}$ cells. Doxorubicin $IC_{50}$ and accumulation data were obtained as described for SW-1573/1R500 cells in Figure 1. ○, MCF- $7^{ADR}$ minus verapamil; ●——●, MCF-$7^{ADR}$ plus verapamil; □, the parent cell line MCF-7. Data shown are means ± s.e. for two independent cytotoxicity and accumulation experiments each carried out in duplicate and triplicate, respectively. Correlation coefficient is 0.997 for data obtained with 8–128 ±M verapamil and 0.98 with 0–128 $\mu$M verapamil. However, accumulation values at 0 $\mu$M verapamil likely have been overestimated as a result of excessive sticking of doxorubicin to the cells at the very high (290 $\mu$M) concentration used. Dx, doxorubicin; Vp, verapamil.

**Figure 3** Verapamil-induced stimulation of doxorubicin accumulation in MCF-$7^{ADR}$ cells. Doxorubicin accumulation was measured on trypsinised MCF-$7^{ADR}$ and MCF-7 cells as described under Materials and methods at a fixed concentration of doxorubicin (0.5 μM) after a 2 h incubation period as a function of the verapamil concentration. ●——●, MCF-$7^{ADR}$, O——O, MCF-7. Shown is a particular experiment in which each point represents mean ± s.d. of triplicate samples. Vp, verapamil.

**Figure 4** Relationship between log$IC_{50}$ and cellular doxorubicin at $IC_{50}$ in revertant SW-1573/1R500-0 cells. Doxorubicin $IC_{50}$ and accumulation data were obtained as described for SW-1573/1R500 cells in Figure 1. Open symbols: no verapamil present; closed symbols: with verapamil. ∇, ▼: SW-1573/1R500-0 cells; O, ●: the parent cell line SW-1573. For SW-1573 cells only 32 μM verapamil was used. Data are means ± s.d. of three independent experiments each performed in duplicate or triplicate. Correlation coefficient is 0.989 for data obtained with 0–32 μM verapamil in SW-1573/1R500-0 cells. Broken curved line indicates possible extrapolation to sensitive (SW-1573) coordinates. Dx, doxorubicin; Vp, verapamil.

polated straight lines, which would predict results obtained with infinitely high concentrations of verapamil, did not cross the coordinates found for the sensitive cell lines (Figures 1 and 2).

The revertant cell line SW-1573/1R500-0, used because of its intermediate level of doxorubicin resistance (24-fold), shows similar characteristics (Figure 4). The use of 128 μM verapamil resulted in a residual level of doxorubicin resistance of a factor 2 (Figure 4). For this cell line, however, deviations from linearity in the semilogarithmic plot occurred at higher (8–128 μM) verapamil concentrations (see broken curve).

When the data shown in the Figures 1, 2 and 4 were plotted on a linear scale, part of the curve that describes the remaining doxorubicin resistance at high verapamil concentrations turned out to be a straight line (Figures 5 and 6). Strikingly, extrapolation to complete reversal of resistance showed that the line now almost crosses the origin as well as the coordinates of the sensitive SW-1573 and MCF-7 cells (Figures 5a and b, respectively). Thus at least the last part of reversal of resistance can be described by the simple function

$$IC_{50} = c.[dox_i \text{ at } IC_{50}]$$

in which c is a constant and $dox_i$ is the cellular amount of doxorubicin at each $IC_{50}$ value. In SW-1573 variants that show a MDR phenotype without overexpression of P-glycoprotein (Baas *et al.*, 1990; Kuiper *et al.*, 1990) the same relationship was found (Figure 6).

The minimal cellular amount of doxorubicin necessary to reach $IC_{50}$ was 8 pmol $10^{-6}$ cells as found for SW-1573 cells in the presence of verapamil (see Figure 6). Since the majority of doxorubicin in sensitive cells is present in the nucleus (Seeber *et al.*, 1980), the nuclear amount of doxorubicin necessary to reach $IC_{50}$ was approximately 8 pmol $10^{-6}$ cells.

For the SW-1573/1R500-0 cells the curve did not cross the coordinates found for the sensitive SW-1573 cells (Figure 6). When, however, the intracellular doxorubicin amounts at $IC_{50}$ were corrected for the cytoplasmic volume (which is relatively large in this cell line; see Table I) in a way described under Materials and methods and in the legends of Figure 6, a corrected line was obtained which now closely approaches the coordinates of the sensitive cell line. Only small corrections due to differences in cellular volume between sensitive and resistant cells were necessary for the other cell lines including MCF-7 cells (Table I). Corrected lines are therefore not shown in the Figures.

*Doxorubicin N/C fluorescence ratios in sensitive and MDR cells*

Previously we have presented evidence that the increased amounts of intracellular doxorubicin at $IC_{50}$ in MDR cells and the verapamil-induced reversal of this process results from changes in intracellular distribution of doxorubicin (Schuurhuis *et al.*, 1989*a*). We now have measured similar resistance-related and/or verapamil-induced changes in the ratio nuclear doxorubicin fluorescence and cytoplasmic doxorubicin fluorescence in human MDR cancer cell lines (Table II). Figure 7 illustrates doxorubicin fluorescence distribution in the 2780 ovarian cancer cell lines in the presence of different concentrations of verapamil. In parental A2780 cells relatively little fluorescence is present in the cytoplasm (Figure 7a). This situation is not altered when verapamil is used (Figure 7b). In contrast, resistant $2780^{AD}$ cells contain a relatively large fraction of the total cellular fluorescence in the cytoplasm, either as a diffuse Golgi-like cloud or in a punctuate pattern throughout the cytoplasm (Figure 7c). Verapamil in increasing concentrations increases the percentage of cells with preferential nuclear fluorescence localisation (compare situation in the absence of verapamil in Figure 7c with d and e, which illustrate the effects of 4 and 8 μM verapamil, respectively). In the presence of 128 μM of verapamil almost all cells showed preferential nuclear fluorescence (illustrated in Figure 7f). The situation was not completely the same as in the sensitive cell line, since residual punctuate cytoplasmic fluorescence was still present. Qualitatively similar results were found for the other cell lines used (not shown here). Of particular interest doxorubicin fluorescence distribution in SW-1573/1R50 cells, which do not overexpress P- glycoprotein (Baas *et al.*, 1990), could not be modulated with verapamil similar to the situation for other non-P-glycoprotein/MDR cell lines (Schuurhuis *et al.*, 1991).

*Nuclear amounts of doxorubicin at equitoxic concentrations*

In Figure 8 it is shown that intracellular doxorubicin amounts at $IC_{50}$ are increased in the MDR Chinese hamster cells and can be decreased by verapamil in a dose-dependent way as shown in this paper for other MDR cells. The photographs in Figure 9 further illustrate that this is most probably largely due to a verapamil-reversible increase of cytoplasmic doxorubicin during development of resistance. On

**Figure 5** Relationship between $IC_{50}$ and cellular doxorubicin at $IC_{50}$ in SW-1573/1R500 and MCF-$7^{ADR}$ cells. Data from the Figures 1 and 2 were replotted on a linear scale in Figure 5a (SW-1573/1R500 and SW-1573) and Figure 5b (MCF-$7^{ADR}$ and MCF-7), respectively. For Figure 5a correlation coefficients were 0.999 for data obtained with 8–128 μM verapamil. The same was found when the data for the parent cell line SW-1573 and the origin (0,0) were included. O, SW-1573/1R500 minus verapamil; ●——●, SW-1573/1R500 plus verapamil; □, the parent cell line SW-1573. for Figure 5b the correlation coefficient was 0.997 for data obtained with 32–128 μM verapamil. The same was found when the data for the parent cell line MCF-7 and the origin were included. O, MCF-$7^{ADR}$ minus verapamil; ●——●, MCF- $7^{ADR}$ plus verapamil; □, the parent line MCF-7. Dx, doxorubicin; Vp, verapamil.

the other hand the photographs in Figure 9 also illustrate that the *nuclear* doxorubicin fluorescence, measured at equitoxic concentrations, may vary much less over the whole range of reversal of resistance. As a result of these processes total cellular fluorescence was much higher in $CH^RC5$ cells without verapamil than in AUXB1 cells without verapamil (factor >13), when measured under equitoxic conditions (700 μM and 1 μM, respectively, corresponding to the Figures 9f and a, respectively) and when using the method that enabled direct comparison of fluorescence signals (see Materials and methods under 'quantification of drug distribution with laser scan microscopy'). This difference was reduced to a factor 9.7 ± 4.3 ($n = 3$) under conditions illustrated in Figure 9e. A further reduction to 2.7 ± 1.4 ($n = 3$) was found using 8 μM doxorubicin plus 4 μM verapamil (Figure 9d). At complete reversal of resistance total cellular fluorescence was only a factor 1.2 ± 0.3 ($n = 3$) higher than in AUXB1 cells.

No quenching of cytoplasmic fluorescence has been assumed to occur (see Materials and methods; Tarasiuk *et al.*, 1989). Recently, however, a possible concentration-dependent quenching of daunorubicin in denucleated cells has been reported (Slapak *et al.*, 1992). Consequently, at the higher drug concentrations (e.g. >3 μM in Figure 8), C-values may have been underestimated. The measured N/C ratios and thereby the calculated N-values may thus have been overestimated. This would fit even better with the conclusion that N-values are constant irrespective residual resistance levels.

Unfortunately, determination of N/C ratios was inaccurate especially at higher levels of resistance because part of the cytoplasmic fluorescence overlapped the nuclear fluorescence. As a result the N value in the N/C ratio, and thereby the calculated total nuclear doxorubicin amounts, will be overestimated at the higher levels of resistance. Nevertheless Figure 8 shows that at complete reversal of resistance (32 μM verapamil) the calculated *nuclear* drug amounts are the same as in AUXB1 cells.

### Discussion

From the literature it appears that there is almost consensus about the fact that multidrug resistance is multifactorial. For example, the factors mentioned to contribute to anthracycline or VP-16 resistance in MDR cells include decreased drug accumulation, caused by changes in drug influx and/or drug efflux (Bradley *et al.*, 1988) and mostly parallelled by changes in the intracellular distribution of the drug (Broxterman *et al.*, 1990; Gervasoni *et al.*, 1991; Gigli *et al.*, 1989; Hindenburg *et al.*, 1987, 1989; Keizer *et al.*, 1989; Schuurhuis *et al.*, 1989*a*, 1991; Willingham *et al.*, 1986), changes in topoisomerase II activity/levels (Beck, 1989) or alterations in drug activation or in the capacity to detoxify reactive drug-induced oxygen-derived free radicals (Sinha & Mimnaugh, 1990). In the present paper we have made an analysis of factors contributing to drug resistance in MDR cells by the use of a quantitative approach.

First we have shown that development of doxorubicin resistance in human lung cancer and human breast cancer cells is characterised by an increase in cellular amounts of drug measured at equitoxic drug concentrations. Apart from this decrease in drug efficacy it is known that these cells have a decreased drug accumulation compared to parent cells when exposed to a fixed external concentration of drug. This is due, at least for a part, to the presence of the putative drug P-glycoprotein efflux pump in these cells (Fairchild *et al.*, 1987; Keizer *et al.*, 1989). The decrease in drug efficacy might in principle be caused by factors such as alterations of glutathione transferase activity as mentioned above. However, an agent like verapamil, known for its potent stimulation of drug accumulation in such P-glycoprotein-containing cells (Bradley *et al.*, 1988; Ford *et al.*, 1988; Keizer *et al.*, 1989; Politi *et al.*, 1990) turned out to be active in reversing the decreased doxorubicin effectiveness as well (Figures 1, 2 and 4–6). As will be explained below it is unlikely that verapamil exerts this action via an effect on other factors

(such as glutathione transferase activity) mentioned earlier to contribute to doxorubicin resistance.

For the high level MDR cell lines it was found that reversal of resistance can be described by a function of the type

$$IC_{50} = e^{c[\text{doxi at IC50}]}$$

in which c is a constant.

This relationship is applicable to moderate to high levels of residual resistance, i.e. resistance left upon verapamil exposure. At low to moderate residual levels of resistance the data fit into a simpler function:

$$IC_{50} = c.[\text{dox}_i \text{ at } IC_{50}]$$

in which c is a constant.

In Figure 10 a simple model is depicted which may explain the relationships found between $IC_{50}$ and [$dox_i$ at $IC_{50}$]. If

**Figure 6** Relationship between $IC_{50}$ and cellular doxorubicin at $IC_{50}$ in SW-1573/1R500-0, SW-1573/1R50 and SW-1573/2R30 cells. Data from Figure 4 were replotted on a linear scale. In addition, data obtained for the non-P-glycoprotein/MDR cell lines SW-1573/2R30 and SW-1573/1R50 are added. Open symbols, no verapamil present; closed symbols, plus verapamil (32 μM in SW-1573, SW-1573/1R50 and SW-1573/2R30 cells). ∇, ▼: SW-1573/1R500-0 cells; □, ■: SW-1573/1R50 cells; Δ, ▲: SW-1573/2R30 cells; O, ●: parental SW-1573 cells. The correlation coefficient was >0.99 for SW-1573/1R500-0 cells for data obtained with 2–128 μM verapamil, also when the origin was included. The same was found for SW-1573/1R50 and SW-1573/2R30 cells. Note that coordinates of origin, SW-1573 cells minus verapamil (O) and the non-P-glycoprotein/MDR cells (□, Δ) indicate a linear relationship between $IC_{50}$ and cellular doxorubicin at $IC_{50}$ in SW-1573/2R30 but not in SW-1573/1R50 cells.

The data obtained for SW-1573/1R500-0 cells when cellular amounts of doxorubicin had been corrected for cytoplasmic volume are shown too (◆—◆; r>0.99, when now also the coordinates of SW-1573 cells were included). Corrections were made using the formula shown under Materials and methods. It is assumed that nuclear amounts of doxorubicin are the same at all $IC_{50}$ values found for SW-1573 and SW-1573/1R500-0 cells, which is highly likely as explained in the Results section under 'nuclear amounts of doxorubicin at equitoxic concentrations'. For reference to residual levels of resistance in the highly resistant cell line SW-1573/1R500, the last part of Figure 5a (at doxorubicin concentrations <1 μM) is indicated (——····). Dx, doxorubicin; Vp, verapamil. In the inset it is shown that at verapamil concentrations <2 μM deviations from linearity occur in SW-1573/IR500-00 cells similar to those seen at low verapamil concentrations in Figure 5.

reversal of resistance by verapamil would have been caused solely by stimulation of drug accumulation, [$dox_i$ at $IC_{50}$] would have been constant in the presence or absence of different concentrations of verapamil (line I in Figure 10A). On the other hand, if hypothetically the verapamil-induced reversal of resistance would not be parallelled at all by a stimulation of drug accumulation, [$dox_i$ at $IC_{50}$] should be lower in the presence of verapamil (line II in Figure 10A). With this in mind the shape of the curves as shown in the Figures 5 and 6 can easily be explained assuming that verapamil shows both effects at residual resistance levels of >17 (SW-1573/1R500), >6 (MCF-$7^{ADR}$) and >7.5 (SW-1573/1R500-0), while at residual resistance levels of 7–17 (SW-1573/1R500), 4–6 (MCF-$7^{ADR}$) and 2–7.5 (SW-1573/1R500-0) it acts almost exclusively via its accumulation-independent effect.

In the 7.4-fold resistant SW-1573/1R50 cells, which do not express P-glycoprotein (Baas *et al.*, 1990), an accumulation defect of 1.6 was found (not shown), indicating that *emergence* of resistance in these cells occurred both via a decrease of drug accumulation and a decrease of drug efficacy. This is compatible with the observation that a curve which would include the coordinates of the origin, SW-1573 (minus verapamil) and SW-1573/1R50 (minus verapamil) (see Figure 6) would be intermediate between lines I and II in Figure 10B. In contrast, the coordinates of origin, SW-1573 cells minus verapamil and SW-1573/2R30 cells minus verapamil (Figure 6) indicate a close-to- linear relationship between $IC_{50}$ and [$dox_i$ at $IC_{50}$]. This shows that a change of doxorubicin efficacy may be the main or only factor responsible for the low degree of doxorubicin resistance in these cells

**Figure 7** Subcellular doxorubicin fluorescence distribution in 2780 human ovarian cancer cells in the presence of different concentrations of verapamil. A2780 parental and $2780^{AD}$MDR cells were incubated for 2 h at 37°C with doxorubicin and with different concentrations of verapamil. In order to improve detection of fluorescence in nucleus and cytoplasm, doxorubicin concentrations were used that resulted in about equal intracellular amounts at all combinations used. **a**, A2780; 4 μM doxorubicin, no verapamil; **b**, A2780; 4 μM doxorubicin, 32 μM verapamil. **c**, $2780^{AD}$, 20 μM doxorubicin, no verapamil. **d**, $2780^{AD}$; 15 μM doxorubicin, 4 μM verapamil. **e**, $2780^{AD}$; 10 μM doxorubicin, 8 μM verapamil. **f**, $2780^{AD}$; 5 μM doxorubicin, 32 μM verapamil. Note mainly nuclear localisation of fluorescence in **a**, **b**, **e** and **f** and mainly cytoplasmic localisation of fluorescence in **c** and **d**. Incidentally cells with mainly nuclear localisation can be observed under conditions illustrated in **d** (arrowhead); incidentally cells with mainly cytoplasmic fluorescence localisation can be seen under conditions illustrated in **e** (arrowhead). Bar in **a**, indicates 10 μm.

**Table I** Cellular and nuclear diameters of MDR and sensitive cells

| Cell line | d cell (μm) | d nucleus (μm) | Calculated cytopl. vol. (μm³) |
|---|---|---|---|
| SW-1573 | 16.15 ± 1.20[a](9)[b] | 10.47 ± 0.84 (3) | 1606 |
| SW-1573/1R500 | 16.82 ± 1.31 (9) | 10.54 ± 0.97 (4) | 1877 |
| SW-1573/1R500-0 | 17.92 ± 0.70[c](7) | 10.54 ± 1.54 (2) | 2399 |
| AUXB1 | 12.96 ± 0.31 (4) | – | – |
| $CH^RC_5$ | 13.69 ± 1.35 (4) | – | – |
| MCF-7 | 16.20 ± 1.64 (4) | – | – |
| MCF-7$^{ADR}$ | 16.41 ± 0.83 (4) | – | – |

[a]Mean ± s.d. [b]Values in parenthesis indicate number of independent experiments. [c]Significantly different from SW-1573 value ($P < 0.01$, Student's $t$-test). –, not determined.

**Table II** Subcellular doxorubicin fluorescence distribution in human breast, lung and ovarian cancer MDR and sensitive cell lines

| Cell line | Modifier (μM) | $IC_{50}$[a] (μM) | [$dox_i$ at $IC_{50}$[a]] (pmol $10^{-6}$ cells) | N/C ratio[b] |
|---|---|---|---|---|
| MCF-7 | None | 0.19 | 16 | exp.1: 3.6 ± 1.1 |
| | | | | exp.2: 3.2 ± 0.9 |
| MCF-7$^{ADR}$ | None | 290 | 3542 | exp.1: 0.1 ± 0.1 |
| | | | | exp.2: 0.1 ± 0.1 |
| | 16 Vp | 1.8 | 99 | exp.1: 0.8 ± 0.3 |
| | 128 Vp | 0.75 | 46 | exp.1: 2.5 ± 0.8 |
| | | | | exp.2: 1.3 ± 0.7 |
| SW-1573 | None | 0.11 | 13.5 | 2.8 ± 0.1 ($n$ = 3) |
| SW-1573/1R500 | None | 38 | 490 | 0.3 ± 0.3 |
| | 8 Vp | 2 | 220 | 1.7 ± 1.5 |
| | 128 Vp | 0.75 | 80 | 2.2 ± 1.0 |
| SW-1573/1R500-0 | None | 2.6 | 448 | 1.2 ± 0.7 |
| | 128 Vp | 0.21 | 43 | 2.5 ± 1.1 |
| SW-1573/1R50 | None | 0.78 | 63 | 1.9 ± 0.1* ($n$ = 4) |
| | 32 Vp | 0.37 | 28 | 1.8 ± 0.1 |
| A2780 | None | 0.24 | 24 | 2.9 ± 1.2 |
| 2780$^{AD}$ | None | 17.4 | 128 | 0.4 ± 0.2 |
| | 32 Vp | 0.45 | 35 | 2.7 ± 1.0 |

[a]Mean of 2–7 independent experiments (s.s. < 20%). [b]Mean (± s.s.) of at least 30–50 cells in a representative experiment, except in the case of MCF-7 and MCF-7$^{ADR}$ cells (two independent experiments as indicated; each 30–50 cells) and SW-1573 and SW-1573/1R50 cells (given are M ± SD of 3–4 independent experiments). *Significantly different from N/C ratio of SW-1573 parent cells ($P < 0.01$, Student's $t$-test).

N/C ratios were determined at $IC_{50}$ values of doxorubicin except for the sensitive cells or when verapamil (Vp) was used (in those cases 2 μM doxorubicin was used since at lower concentrations background noise became too high). N/C ratios are independent of doxorubicin concentration in a large range of drug concentrations (De Lange *et al.*, 1992), suggesting that this is also the case at concentrations lower than 2 μM.

(compare with line II in Figure 10A and B. This has been confirmed in accumulation experiments performed at a fixed (0.5 μM) doxorubicin concentration which revealed no accumulation defect (not shown).

Continuous exposure of SW-1573/2R30 cells to doxorubicin led to isolation of SW-1573/2R50 cells, which are of a non-P- glycoprotein MDR phenotype (Baas *et al.*, 1990; Kuiper *et al.*, 1990) and after prolonged exposure to cells with an increased *mdr*-1 mRNA and P-glycoprotein expression (Kuiper *et al.*, 1990). Similar results have been found for murine erythroleukaemia cells (Slapak *et al.*, 1990). It could thus be that doxorubicin resistance in low level MDR non-P-glycoprotein lung cancer cells as well as doxorubicin resistance of early selected cells of the high level MDR P-glycoprotein cell lines MCF-7$^{ADR}$ and 2780$^{AD}$, is caused, at least partly, by the same P-glycoprotein-*independent* phenotypic change that occurs in P-glycoprotein-mediated resistance, i.e. a change in intracellular drug efficacy, which would, at least partly, be caused by a change in subcellular drug distribution.

In the present study it is shown that a partial reversal of doxorubicin resistance was accomplished in human MDR cells which was accompanied by a partial reversal of N/C ratio changes (Table II). Complete reversal of doxorubicin resistance in $CH^RC5$ P-glycoprotein/MDR Chinese hamster ovarian cells to parental AUXB1 levels was accompanied by a complete reversal of the drug accumulation defect as well as the drug distribution change (Schuurhuis *et al.*, 1989*a*). The consequence of these findings would be that it is mainly if not only the nuclear *concentration* of drug which determines sensitivity/resistance. For $CH^RC_5$ cells we confirmed this relationship (Figures 8, 9). It cannot be excluded, however, that verapamil acts via a mechanism comprising increment of *nuclear* doxorubicin efficacy. This mechanism of resistance reversal might prevail in non-P-glycoprotein MDR cells. The fact that considerable decrease of doxorubicin resistance in such cells by verapamil is possible with only small concomitant changes of drug accumulation or drug distribution (this paper; Schuurhuis *et al.*, 1991), are in favour of this idea. The results presented in the present paper for P-glycoprotein MDR cells are nevertheless in agreement with a paper of Gigli and colleagues, who did not study verapamil effects, but, making use of spectral properties of doxorubicin, showed that nuclear concentrations of doxorubicin were not significantly different at equitoxic drug concentrations in sensitive and resistant K562 human leukaemia cells (Gigli *et al.*, 1989). Also, in a study comparing cellular doxorubicin accumulation, cytotoxicity and DNA lesions in sensitive and multidrug-resistant human myeloma cells, Bellamy *et al.* showed that at low doxorubicin concentrations important for cytotoxicity, equal *cellular* concentrations in both sensitive and resistant cells, obtained by adjustment of extracellular drug concentrations, nevertheless caused less cytotoxicity in resistant cells (Bellamy *et al.*, 1988*b*). DNA

**Figure 8** Interrelationship between $IC_{50}$, cellular doxorubicin at $IC_{50}$ and nuclear doxorubicin at $IC_{50}$ in adherent $CH^RC5$ and AUXB1 cells. $IC_{50}$ values were obtained in an assay including a 2 h incubation with doxorubicin as described under Materials and methods. Total *cellular* amounts of doxorubicin at $IC_{50}$ obtained with different concentrations of verapamil were determined in a 2 h incubation assay using [$^{14}$C]-labelled doxorubicin as described under Materials and methods. *Nuclear* amounts of doxorubicin were calculated from N/C fluorescence ratio measurements with laser scan microscopy (corrected for fluorescence quenching by DNA as described under Materials and methods) and from the accumulation experiments described above (giving total cellular amounts of doxorubicin). Open symbols: measured cellular amounts of doxorubicin in $CH^RC5$ cells (○——○) and AUXB1 cells (□); closed symbols: calculated nuclear amounts of doxorubicin in $CH^RC5$ cells (● --- ●) and AUXB1 cells (■). Doxorubicin amounts are in pmol $10^{-6}$. No verapamil was used for AUXB1 cells. Data are means ± s.d. from 3–9 independent cytotoxicity, accumulation and N/C fluorescence ratio measurements. The numbers **a**, **c-f** refer to the photographs shown in Figure 9.

damage in the resistant cells was also less under these conditions (compare Bellamy *et al.*, 1988*a* and *b*). This is in good agreement with the observation that N/C ratios are lower in these resistant cells (De Lange *et al.*, 1992; Broxterman *et al.*, 1990). This suggests, as outlined extensively in the present paper, that higher *cellular* concentrations are needed in the resistant cells in order to obtain the same *nuclear* concentrations as in sensitive cells.

It has to be stated that other reversing agents behave in a way similar to verapamil: bepridil, Ro 11-2933/001 and Cremophor EL have been tested as to this (Schuurhuis *et al.*, 1989*a*, 1990*a*). None of the modifiers, even at high concentrations, was able to overcome completely accumulation defects in human MDR cells (see e.g. Schuurhuis *et al.*, 1990*b*). Verapamil has been used mainly because it is less toxic than several other reversing agents and because it is the most widely studied reverter.

**Figure 9** Subcellular doxorubicin fluorescence distribution in the presence or absence of verapamil at equitoxic concentrations in $CH^RC5$ and AUXB1 cells. Adhered cells were incubated for 2 h at 37°C with combinations of different concentrations of doxorubicin and verapamil, as specified below, which all resulted in the same growth-inhibition. Doxorubicin concentrations used were in all cases 2.5 times higher than the $IC_{50}$ values, shown in Figure 7, since otherwise the fluorescence in nucleus and/or cytoplasm could hardly be visualised and quantified. Viability as determined with the trypan blue exclusion test remained >99% for at least 12 h when cells were incubated without any drug present after the 2 h period in the presence of doxorubicin and/or verapamil. **a** and **c–f**: fluorescence microscopy; **b**: phase contrast microscopy. **a**, AUXB1, 1 μM doxorubicin without Vp. **b**, AUXB1, same cells as in **a**, phase contrast. **c**, $CH^RC5$, 1 μM doxorubicin plus 32 μM Vp. **d**, $CH^RC5$, 8 μM doxorubicin plus 4 μM Vp. **e**, $CH^RC5$, 50 μM doxorubicin plus 2 μM Vp. **f**, $CH^RC5$, 700 μM doxorubicin without Vp. N, nucleus; C, cytoplasm; Dx, doxorubicin; Vp, verapamil. Bar in **a** indicates 20 μm.

**Figure 10** Theoretical relationship between $IC_{50}$ and cellular doxorubicin measured at $IC_{50}$ assuming that two factors contribute to doxorubicin resistance and that two modes of action of verapamil exist. **A**, Reversal of resistance by verapamil occurs solely by stimulation of drug accumulation (I) or solely by changing the efficacy of cellular doxorubicin (II). **B**, Emergence of resistance occurs solely by a decrease of drug accumulation (I) or solely by changes in efficacy of cellular doxorubicin (II).

Apart from MDR Chinese hamster ovarian (this paper; Schuurhuis *et al.*, 1989*a*), human ovarian cancer (this paper; Schuurhuis *et al.*, 1989*a*), human lung cancer (this paper; Keizer *et al.*, 1989) and human breast cancer (this paper; Gervasoni *et al.*, 1991) cells, also P-glycoprotein containing KB (Willingham *et al.*, 1986), human myeloma (Broxterman *et al.*, 1990) and human leukaemia (Gigli *et al.*, 1989) cells show changes in subcellular drug distribution in addition to changes in cellular drug accumulation. It is therefore tempting to speculate that the relatively simple explanation for doxorubicin resistance in P-glycoprotein/MDR cells, including the two factors depicted in this paper, is generally applicable.

It has been speculated that changes in free radical detoxifying enzymes such as glutathione transferase and glutathione peroxidase contribute to doxorubicin resistance in P-glycoprotein/MDR MCF-$7^{ADR}$ cells (Batist *et al.*, 1986; Cowan *et al.*, 1986; Sinha *et al.*, 1987; Sinha & Mimnaugh, 1990). From our results, however, it appears that also in MCF-7 MDR cells decreases in drug accumulation together with changes in drug distribution can largely account for doxorubicin resistance. This conclusion is based on proven effects of verapamil on drug accumulation and drug distribution. We have found no anomalous effects of verapamil on glutathione transferase, which might have coincided with resistance reversal: this enzyme (48-fold increased in MCF-$7^{ADR}$ in our hands) was not affected by verapamil concentrations as high as 1 mM (not shown). Such verapamil concentrations are beyond those calculated to occur in cells using effective verapamil concentrations (Broxterman *et al.*, 1988; Cano-Gauci & Riordan, 1987; Yusa & Tsuruo, 1989). Verapamil also had no effect on glutathione peroxidase activity (Batist *et al.*, 1991). Lastly, it has been found that transfection of the gene encoding for glutathione transferase did not confer resistance to doxorubicin (Moscow *et al.*, 1989). Since changes in expression of enzymes suggested to be involved in detoxification of xenobiotics apparently are not involved in doxorubicin resistance we speculate that transfection of the gene encoding for glutathione peroxidase will not confer resistance to doxorubicin in MCF-7 or other parental cell lines.

Upon the mechanism by which MDR cells acquire an altered subcellular drug distribution can only be speculated. Small amounts of P-glycoprotein present on the membranes of cytoplasmic vesicles (Willingham *et al.*, 1987) might force the drug into the vesicles. Alternatively, a drop in vesicular pH, probably parallelling an increase of the cytoplasmic pH under certain conditions (Keizer & Joenje, 1989; Thiebaut *et al.*, 1990) or an increase of the vesicular compartment (Sehested *et al.*, 1987) might explain higher drug accumulation in cytoplasmic compartments, resulting in decreased N/C ratios.

In conclusion, the results presented in this paper show that, at least in P-glycoprotein MDR cells, changes in drug accumulation together with changes in subcellular drug distribution largely account for doxorubicin resistance, thereby excluding the need to postulate additional mechanisms for these cell lines.

We thank J.-K. Eekman, H. Dekker (Department of Medical Oncology) and F. Rodriguez (Department of Hematology) for technical assistance and Dr A.W.M. Nieuwint for the gift of SW-1573/2R30 cells. This study was supported by grants from The Netherlands Cancer Foundation (grant IKA 88-22), The Bristol-Myers Squibb Company, The Preventiefonds (grant nr. 28/834) and the Division of Hematology and Medical Oncology, Valencia, Spain.

**Abbreviations:** DMF, dose modifying factor = $IC_{50}$ minus resistance modifier/$IC_{50}$ plus resistance modifer; Dx, doxorubicin; $IC_{50}$ = drug concentration, which inhibits cell growth by 50% of control values; MDR, multidrug resistant/resistance; Vp, verapamil.

### References

BAAS, F., JONGSMA, A.P.M., BROXTERMAN, H.J., ARCECI, R.J., HOUSMAN, D., SCHEFFER, G.L., RIETHORST, A., VAN GRONIGEN, M., NIEUWINT, A.W.M. & JOENJE, H. (1990). Non-P-glycoprotein mediated mechanism for multidrug resistance precedes P- glycoprotein expression during *in vitro* selection for doxorubicin resistance in a human lung cancer cell line. *Cancer Res.*, **50**, 5392–5398.

BATIST, G., SCHECTER, R., WOO, A., GREENE, D. & LEHNERT, S. (1991). Glutathione depletion in human and rat multi-drug resistant breast cancer cell lines. *Biochem. Pharmacol.*, **41**, 631–635.

BATIST, G., TULPULE, A., SINHA, B.K., KATKI, A.G., MYERS, C.E. & COWAN, K.H. (1986). Overexpression of a novel anionic glutathione transferase in multidrug-resistant human breast cancer cells. *J. Biol. Chem.*, **261**, 15544–15549.

BECK, W.T. (1989). Unknotting the complexities of multidrug resistance: the involvement of DNA topoisomerases in drug action and resistance. *J. Natl Cancer Inst.*, **81**, 1683–1685.

BELLAMY, W.T., DALTON, W.S., KAILEY, J.M., GLEASON, M.C., MCCLOSKEY, T.M., DORR, R.T. & ALBERTS, D.S. (1988*a*). Verapamil reversal of doxorubicin resistance in multidrug-resistant human myeloma cells and association with drug accumulation and DNA damage. *Cancer Res.*, **48**, 6365–6370.

BELLAMY, W.T., DORR, R.T., DALTON, W.S. & ALBERTS, D.S. (1988*b*). Direct relation of DNA lesions in multidrug- resistant human myeloma cells to intracellular doxorubicin concentration. *Cancer Res.*, **48**, 6360–6364.

BRADLEY, G., JURANKA, P.F. & LING, V. (1988). Mechanism of multidrug resistance. *Biochim. Biophys. Acta*, **948**, 87–128.

BROXTERMAN, H.J., PINEDO, H.M., KUIPER, C.M., KAPTEIN, L.C.M., SCHUURHUIS, G.J. & LANKELMA, J. (1988). Induction by verapamil of a rapid increase in ATP consumption in multidrug-resistant tumor cells. *FASEB J.*, **2**, 2278–2282.

BROXTERMAN, H.J., SCHUURHUIS, G.J., LANKELMA, J., BAAK, J.P.A. & PINEDO, H.M. (1990). Towards functional screening for multidrug resistant cells in human malignancies. In: Mihich, E. (ed.), *Drug Resistance: Mechanism and Reversal*, pp. 309–319. John Libbey: CIC, Roma.

CANO-GAUCI, D.F. & RIORDAN, J.R. (1987). Action of calcium antagonists on multidrug resistant cells. Specific cytotoxicity independent of increased cancer drug accumulation. *Biochem. Pharmacol.*, **36**, 2115–2123.

COLE, S.P.C., CHANDA, E.R., DICKE, F.P., GERLACH, J.H. & MIRSKI, S.E.L. (1991). Non-P-glycoprotein-mediated multidrug resistance in a small cell lung cancer cell line: evidence for decreased susceptibility to drug-induced DNA damage and reduced levels of topoisomerase II. *Cancer Res.*, **51**, 3345–3352.

COLE, S.P.C., DOWNES, H.F. & SLOVAK, M.L. (1989). Effect of calcium antagonists on the chemosensitivity of two multidrug- resistant human tumour cell lines which do not overexpress P- glycoprotein. *Br. J. Cancer*, **59**, 42–46.

COLEY, H.M., WORKMAN, P. & TWENTYMAN, P.R. (1991). Retention of activity by selected anthracyclines in a multidrug resistant human large cell lung carcinoma line without P- glycoprotein hyperexpression. *Br. J. Cancer*, **63**, 351–357.

COWAN, K.H., BATIST, G., TULPULE, A., SINHA, B.K. & MYERS, C.E. (1986). Similar biochemical changes associated with multidrug resistance in human breast cancer cells and carcinogen- induced resistance to xenobiotics in rats. *Proc. Natl Acad. Sci. USA*, **83**, 9328–9332.

DANKS, M.K., YALOWICH, J.C. & BECK, W.T. (1987). Atypical multidrug resistance in a human leukemic cell line selected for resistance to tenoposide (VM-26). *Cancer Res.*, **47**, 1297–1301.

DE LANGE, J.H.M., SCHIPPER, N.W., SCHUURHUIS, G.J., TEN KATE, T.K., VAN HEIJNINGEN, TH.H.M., PINEDO, H.M., LANKELMA, J. & BAAK, J.P.A. (1992). Quantification of intracellular doxorubicin distribution in multidrug resistant and sensitive cells by laserscan microscopy and digital image processing. *Cytometry*, **13**, 572–576.

FAIRCHILD, C.R., IVY, S.P., KAO-SHAN, C.-S., WHANG-PENG, J., ROSEN, N., ISRAEL, M.A., MELERA, P.W., COWAN, K.H. & GOLDSMITH, M.E. (1987). Isolation of amplified and overexpressed DNA sequences from adriamycin-resistant human breast cancer cells. *Cancer Res.*, **47**, 5141–5148.

FORD, J.M., PROZIALECK, W.C. & HAIT, W.N. (1988). Structural faetures determining activity of phenothiazines and related drugs for inhibition of cell growth and reversal of multidrug resistance. *Molec. Pharmacol.*, **35**, 105–115.

GERVASONI, J.E., FIELDS, S.Z., KRISHNA, S., BAKER, M.A., ROSADO, M., THURAISAMY, K., HINDENBURG, A.A. & TAUB, R.N. (1991). Subcellular distribution of daunorubicin in P-glycoprotein-positive and -negative drug-resistant cell lines using laser-assisted confocal microscopy. *Cancer Res.*, **51**, 4955–4963.

GIGLI, M., RASOANAIVO, T.W.D., MILLOT, J.-M., JEANNSSON, P., RIZZO, V., JARDILLIER, J.-C., ARCAMONE, F. & MANFAIT, M. (1989). Correlation between growth inhibition and intranuclear doxorubicin and 4′-deoxy-4′-iododoxorubicin quantified in living K562 cells by microspectrofluorometry. *Cancer Res.*, **49**, 560–564.

HABER, M., NORRIS, M.D., KAVALLARIS, M., BELL, D.R., DAVEY, R.A., WHITE, L. & STEWART, B.W. (1989). Atypical multidrug resistance in a therapy-induced drug-resistant human leukemia cell line (LALW-2): resistance to vinca alkaloids independent of P-glycoprotein. *Cancer Res.*, **49**, 5281–5287.

HARKER, W.G., SLADE, D.L., DALTON, W.S., MELTZER, P.S. & TRENT, J.M. (1989). Multidrug resistance in mitoxantrone-selected HL-60 leukemia cells in the absence of P-glycoprotein overexpression. *Cancer Res.*, **49**, 4542–4549.

HINDENBURG, A.A., BAKER, M.A., GLEYZER, E., STEWART, V.J., CASE, N. & TAUB, R.N. (1987). Effect of verapamil and other agents on the distribution of anthracyclines and on reversal of drug resistance. *Cancer Res.*, **47**, 1421–1425.

HINDENBURG, A.A., GERVASONI, J.E., KRISHNA, S., STEWART, V.J., ROSADO, M., LUTZKY, J., BHALLA, K., BAKER, M.A. & TAUB, R.N. (1989). Intracellular distribution and pharmacokinetics of daunorubicin in anthracycline-sensitive and -resistant HL-60 cells. *Cancer Res.*, **49**, 4607–4614.

KEIZER, H.G. & JOENJE, H. (1989). Increased cytosolic pH in multidrug-resistant human lung tumor cells: effect of verapamil. *J. Natl Cancer Inst.*, **81**, 706–709.

KEIZER, H.G., SCHUURHUIS, G.J., BROXTERMAN, H.J., LANKELMA, J., SCHOONEN, W.G.E.J., VAN RIJN, J., PINEDO, H.M. & JOENJE, H. (1989). Correlation of multidrug resistance with decreased drug accumulation, altered subcellular drug distribution, and increased P-glycoprotein expression in cultured SW-1573 human lung tumor cells. *Cancer Res.*, **49**, 2988–2993.

KUIPER, C.M., BROXTERMAN, H.J., BAAS, F., SCHUURHUIS, G.J., HAISMA, H.J., SCHEFFER, G.L., LANKELMA, J. & PINEDO, H.M. (1990). Drug transport variants without P-glycoprotein overexpression from a human squamous lung cancer cell line after selection with doxorubicin. *J. Cell Pharmacol.*, **1**, 35–41.

MCGRATH, T. & CENTER, M.S. (1988). Mechanisms of multidrug resistance in HL60 cells. Evidence that a surface membrane protein distinct from P-glycoprotein contributes to reduced cellular accumulation of drug. *Cancer Res.*, **48**, 3959–3963.

MCGRATH, T., MARQUARDT, D. & CENTER, M.S. (1989). Multiple mechanisms of adriamycin resistance in the human leukemia cell line CCRF-CEM. *Biochem. Pharmacol.*, **38**, 497–501.

MOSCOW, J.A., TOWNSEND, A.J. & COWAN, K.H. (1989). Elevation of II class glutathione S-transferase activity in human breast cancer cells by transfection of the GST II gene and its effect on sensitivity to toxins. *Molec. Pharmacol.*, **36**, 22–28.

POLITI, P.M., ARNOLD, S.T., FELSTED, R.L. & SINHA, B.K. (1990). P-glycoprotein-independent mechanism of resistance to VP-16 in multidrug-resistant tumor cell lines: pharmacokinetic and photoaffinity labelling studies. *Mol. Pharmacol.*, **37**, 790–796.

SCHEPER, R.J., BULTE, J.W.M., BRAKKEE, J.G.P., QUAK, J.J., VAN DER SCHOOT, E., BALM, A.J.M., MEIJER, C.J.L.M., BROXTERMAN, H.J., KUIPER, C.M., LANKELMA, J. & PINEDO, H.M. (1988). Monoclonal antibody JSB-1 detects a highly conserved epitope on the P-glycoprotein associated with multi-drug resistance. *Int. J. Cancer*, **42**, 389–394.

SCHUURHUIS, G.J., BROXTERMAN, H.J., CERVANTES, A., VAN HEIJNINGEN, TH.H.M., DE LANGE, J.H.M., BAAK, J.P.A., PINEDO, H.M. & LANKELMA, J. (1989*a*). Quantitative determination of factors contributing to doxorubicin resistance in multidrug-resistant cells. *J. Natl Cancer Inst.*, **81**, 1887–1892.

SCHUURHUIS, G.J., PINEDO, H.M., CERVANTES, A., BROXTERMAN, H.J., VAN KALKEN, C.K., VAN HEIJNINGEN, T.H.H.M. & LANKELMA, J. (1989*b*). Mechanism of anthracycline resistance and its reversal in cells with high and low levels of multidrug resistance. *Proc. Am. Assoc. Cancer Res.*, **30**, 519.

SCHUURHUIS, G.J., BROXTERMAN, H.J., DE LANGE, J.H.M., PINEDO, H.M., VAN HEIJNINGEN, TH.H.M., KUIPER, C.M., SCHEFFER, G.L., SCHEPER, R.J., VAN KALKEN, C.K., BAAK, J.P.A. & LANKELMA, J. (1991). Early multidrug resistance, defined by changes in intracellular doxorubicin distribution, independent of P-glycoprotein. *Br. J. Cancer*, **64**, 857–861.

SCHUURHUIS, G.J., BROXTERMAN, H.J., PINEDO, H.M., VAN HEIJNINGEN, TH.H.M., VAN KALKEN, C.K., VERMORKEN, J.B., SPOELSTRA, E.C. & LANKELMA, J. (1990*a*). The polyoxyethylene castor oil Cremophor EL modifies multidrug resistance. *Br. J. Cancer*, **62**, 591–594.

SCHUURHUIS, G.J., BROXTERMAN, H.J., VAN DER HOEVEN, J.J.M., PINEDO, H.M. & LANKELMA, J. (1987). Potentiation of doxorubicin cytotoxicity by the calcium antagonist bepridil in anthracycline-resistant and -sensitive cell line lines. A comparison with verapamil. *Cancer Chemother. Pharmacol.*, **20**, 285–290.

SCHUURHUIS, G.J., PINEDO, H.M., BROXTERMAN, H.J., VAN KALKEN, C.K., KUIPER, C.M. & LANKELMA, J. (1990*b*). Differential sensitivity of multi-drug-resistant and -sensitive cells to resistance-modifying agents and the relation with reversal of anthracycline resistance. *Int. J. Cancer*, **46**, 330–336.

SEEBER, S., LOTH, H. & CROOKE, S.T. (1980). Comparative nuclear and cellular incorporation of daunorubicin, doxorubicin, carminomycin, marcellomycin, aclacinomycin A and AD32 in daunorubicin-sensitive and -resistant Ehrlich ascites *in vitro*. *J. Cancer Res. Clin. Oncol.*, **98**, 109–118.

SEHESTED, M., SKOVSGAARD, T., VAN DEURS, B. & WINTHER-NIELSEN, H. (1987). Increased plasma membrane traffic in daunorubicin resistant P388 leukaemic cells. *Br. J. Cancer*, **56**, 747–751.

SINHA, B.K., KATKI, A.G., BATIST, G., COWAN, K.H. & MYERS, C.E. (1987). Differential formation of hydroxyl radicals by adriamycin in sensitive and resistant MCF-7 human breast tumor cells: implications for the mechanism of action. *Biochemistry*, **26**, 3776–3781.

SINHA, B.K. & MIMNAUGH, E.G. (1990). Free radicals and anticancer drug resistance: oxygen free radicals in the mechanism of drug cytotoxicity and resistance by certain tumors. *Free Radicals Biology and Medicine*, **8**, 567–581.

SLAPAK, C.A., DANIEL, J.C. & LEVY, S.B. (1990). Sequential emergence of distinct resistance phenotypes in murine erythroleukemia cells under adriamycin selection: decreased anthracycline uptake precedes increased P-glycoprotein expression. *Cancer Res.*, **50**, 7895–7901.

SLAPAK, C.A., LECERF, J.-M., DANIEL, J.C. & LEVY, S.B. (1992). Energy-dependent accumulation of daunorubicin into subcellular compartments of human leukemia cells and cytoplasts. *J. Biol. Chem.*, **267**, 10638–10644.

SLOVAK, M.L., HOELTGE, G.A., DALTON, W.S. & TRENT, J.M. (1988). Pharmacological and biological evidence for differing mechanisms of doxorubicin resistance in two human tumor cell lines. *Cancer Res.*, **48**, 2793–2797.

TARASIUK, J., FREZARD, F., GARNIER-SUILLEROT, A. & GATTEGNO, L. (1989). Anthracycline incorporation in human lymphocytes. Kinetics of uptake and nuclear concentration. *Biochim. Biophys. Acta*, **1013**, 109–117.

TAYLOR, C.W., DALTON, W.S., PARRISH, P.R., GLEASON, M.C., BELLAMY, W.T., THOMPSON, F.H., ROE, D.J. & TRENT, J.M. (1991). Different mechanisms of decreased drug accumulation in doxorubicin and mitoxantrone resistant variants of the MCF7 human breast cancer cell line. *Br. J. Cancer*, **63**, 923–929.

THIEBAUT, F., CURRIER, S.J., WHITAKER, J., HAUGHLAND, R.P., GOTTESMAN, M.M., PASTAN, I. & WILLINGHAM, M.C. (1990). Activity of the multidrug transporter results in alkalinization of the cytosol: measurement of cytosolic pH by microinjection of a pH-sensitive dye. *J. Histochem. Cytochem.*, **38**, 685–690.

WILLINGHAM, M.C., CORNWELL, M.M., CARDARELLI, C.O., GOTTESMAN, M.M. & PASTAN, I. (1986). Single cell analysis of daunomycin uptake and efflux in multidrug-resistant and -sensitive KB cells: effects of verapamil and other drugs. *Cancer Res.*, **46**, 5941–5946.

WILLINGHAM, M.C., RICHERT, N.D., CORNWELL, M.M., TSURUO, T., HAMADA, H., GOTTESMAN, M.M. & PASTAN, I. (1987). Immunocytochemical localization of P170 at the plasma membrane of multidrug-resistant human cells. *J. Histochem. Cytochem.*, **35**, 1451–1456.

YUSA, K. & TSURUO, T. (1989). Reversal mechanism of multidrug resistance by verapamil: direct binding of verapamil to P-glycoprotein on specific sites and transport of verapamil outward across the plasma membrane of K562/ADM cells. *Cancer Res.*, **49**, 5002–5006.

ZAMORA, J.M., PEARCE, H.L. & BECK, W.T. (1988). Physical-chemical properties shared by compounds that modulate multidrug resistance in human leukemic cells. *Mol. Pharmacol.*, **33**, 454–462.