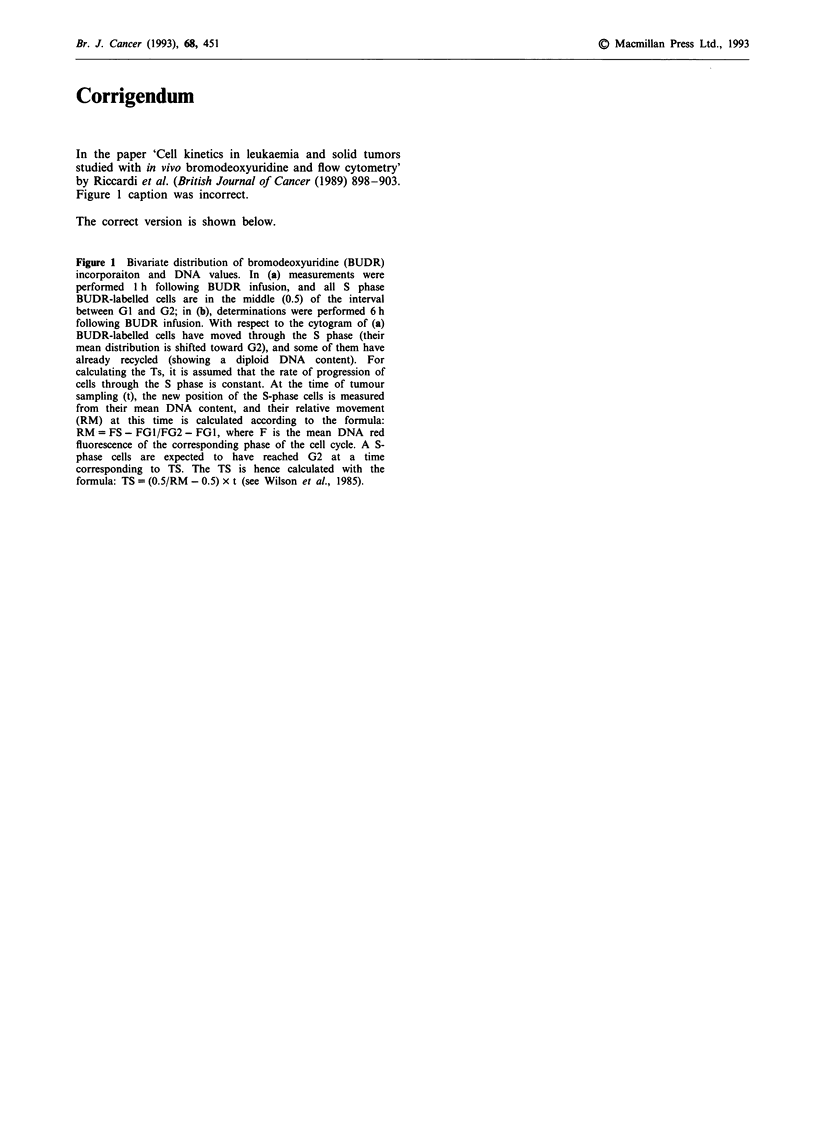# Corrigendum

**Published:** 1993-08

**Authors:** 


 © Macmillan Press Ltd., 1993

### Corrigendum

In the paper 'Cell kinetics in leukaemia and solid tumors studied with *in vivo* bromodeoxyuridine and flow cytometry' by Riccardi *et al.* (*British Journal of Cancer* (1989) 898–903. Figure 1 caption was incorrect.

The correct version is shown below.

**Figure 1** Bivariate distribution of bromodeoxyuridine (BUDR) incorporaiton and DNA values. In (**a**) measurements were performed 1 h following BUDR infusion, and all S phase BUDR-labelled cells are in the middle (0.5) of the interval between G1 and G2; in (**b**), determinations were performed 6 h following BUDR infusion. With respect to the cytogram of (**a**) BUDR-labelled cells have moved through the S phase (their mean distribution is shifted toward G2), and some of them have already recycled (showing a diploid DNA content). For calculating the Ts, it is assumed that the rate of progression of cells through the S phase is constant. At the time of tumour sampling (t), the new position of the S-phase cells is measured from their mean DNA content, and their relative movement (RM) at this time is calculated according to the formula: $RM = FS - FG1/FG2 - FG1$, where F is the mean DNA red fluorescence of the corresponding phase of the cell cycle. A S-phase cells are expected to have reached G2 at a time corresponding to TS. The TS is hence calculated with the formula: $TS = (0.5/RM - 0.5) \times t$ (see Wilson *et al.*, 1985).